# Mirtazapine exerts astrocyte-mediated dopaminergic neuroprotection

**DOI:** 10.1038/s41598-020-77652-4

**Published:** 2020-11-26

**Authors:** Ryo Kikuoka, Ikuko Miyazaki, Natsuki Kubota, Megumi Maeda, Daiki Kagawa, Masaaki Moriyama, Asuka Sato, Shinki Murakami, Yoshihisa Kitamura, Toshiaki Sendo, Masato Asanuma

**Affiliations:** 1 Department of Medical Neurobiology, Okayama University Graduate School of Medicine, Dentistry and Pharmaceutical Sciences, 2-5-1 Shikata-cho, Kita-ku, Okayama, 700-8558 Japan; 2 Department of Clinical Pharmacy, Okayama University Graduate School of Medicine, Dentistry and Pharmaceutical Sciences, Okayama, Japan

**Keywords:** Neurological disorders, Parkinson's disease

## Abstract

Mirtazapine, a noradrenergic and specific serotonergic antidepressant (NaSSA), is known to activate serotonin (5-HT) 1A receptor. Our recent study demonstrated that stimulation of astrocytic 5-HT1A receptors promoted astrocyte proliferation and upregulated antioxidative property in astrocytes to protect dopaminergic neurons against oxidative stress. Here, we evaluated the neuroprotective effects of mirtazapine against dopaminergic neurodegeneration in models of Parkinson’s disease (PD). Mirtazapine administration attenuated the loss of dopaminergic neurons in the substantia nigra and increased the expression of the antioxidative molecule metallothionein (MT) in the striatal astrocytes of 6-hydroxydopamine (6-OHDA)-injected parkinsonian mice via 5-HT1A receptors. Mirtazapine protected dopaminergic neurons against 6-OHDA-induced neurotoxicity in mesencephalic neuron and striatal astrocyte cocultures, but not in enriched neuronal cultures. Mirtazapine-treated neuron-conditioned medium (Mir-NCM) induced astrocyte proliferation and upregulated MT expression via 5-HT1A receptors on astrocytes. Furthermore, treatment with medium from Mir-NCM-treated astrocytes protected dopaminergic neurons against 6-OHDA neurotoxicity, and these effects were attenuated by treatment with a MT-1/2-specific antibody or 5-HT1A antagonist. Our study suggests that mirtazapine could be an effective disease-modifying drug for PD and highlights that astrocytic 5-HT1A receptors may be a novel target for the treatment of PD.

## Introduction

Parkinson’s disease (PD), a progressive neurodegenerative disease, is one of the most prevalent movement disorders worldwide. PD patients exhibit motor symptoms such as akinesia/bradykinesia, resting tremor, rigidity, and postural instability. In the prodromal phase of PD, non-motor symptoms such as constipation, dysosmia, and orthostatic hypotension are observed^1^. Furthermore, mental manifestations such as depression and anxiety are common in the progressive stage of PD^2^. Dopaminergic neuronal loss in the substantia nigra pars compacta (SNpc) is a well-known feature of PD. Motor dysfunction develops when 50% of dopaminergic neuronal terminals and 60–70% of dopamine (DA) in the striatum are lost^3,4^. Therefore, most medications used for PD improve motor dysfunction by restoring dopaminergic activity in the brain. The neuroprotective effects of many candidate agents have been tested in clinical trials^5,6^, but no drugs which slow or stop the progression of PD have been approved thus far.

Oxidative stress is thought to be involved in the pathogenesis of PD. In fact, increased levels of oxidative stress markers such as oxidized lipids, protein, and DNA and decreased levels of the antioxidative molecule glutathione (GSH) have been observed in patients with PD^7,8^. Therefore, reducing oxidative stress by upregulating antioxidant defense could be a promising therapeutic strategy for the treatment of PD.

In the central nervous system, astrocytes protect neurons by releasing neurotrophic factors and the antioxidant molecules GSH and metallothionein (MT)-1/2^9–11^ and by clearing neurotoxic molecules such as excessive glutamate and aggregated α-synuclein^12,13^. We previously reported that the serotonin (5-HT)1A receptor agonist (R)-(+)-8-hydroxy-2-(di-n-propylamino) tetralin hydrobromide (8-OH-DPAT) exerted neuroprotective effects against dopaminergic neurodegeneration by inducing astrocyte proliferation and upregulating MT-1/2 expression in astrocytes^14^. Moreover, we recently reported that the anti-parkinsonian DA agonist rotigotine, which also exhibits 5-HT1A receptor agonistic activity, protects dopaminergic neurons by promoting MT expression in astrocytes^15^. These results suggest that 5-HT1A receptors on astrocytes could be a potential target for dopaminergic neuroprotection.

Mirtazapine is a noradrenergic and specific serotonergic antidepressant which increases the extracellular levels of noradrenaline and serotonin by blocking presynaptic α2 adrenergic receptors on noradrenergic and serotonergic nerve terminals. In addition, mirtazapine inhibits 5-HT2 and 5-HT3 receptors. As a result, mirtazapine indirectly stimulates 5-HT1 receptors, especially 5-HT1A receptors^16^. In this study, to evaluate the potential of mirtazapine as a disease-modifying drug for PD, we explored whether mirtazapine exerted neuroprotective effects against dopaminergic neurotoxin 6-hydroxydopmine (6-OHDA)-induced neurodegeneration by targeting astrocytic 5-HT1A receptors using in vitro and in vivo models of PD.

## Results

### Mirtazapine prevented dopaminergic neurodegeneration via 5-HT1A receptor in parkinsonian mice

**Figure. 1:**
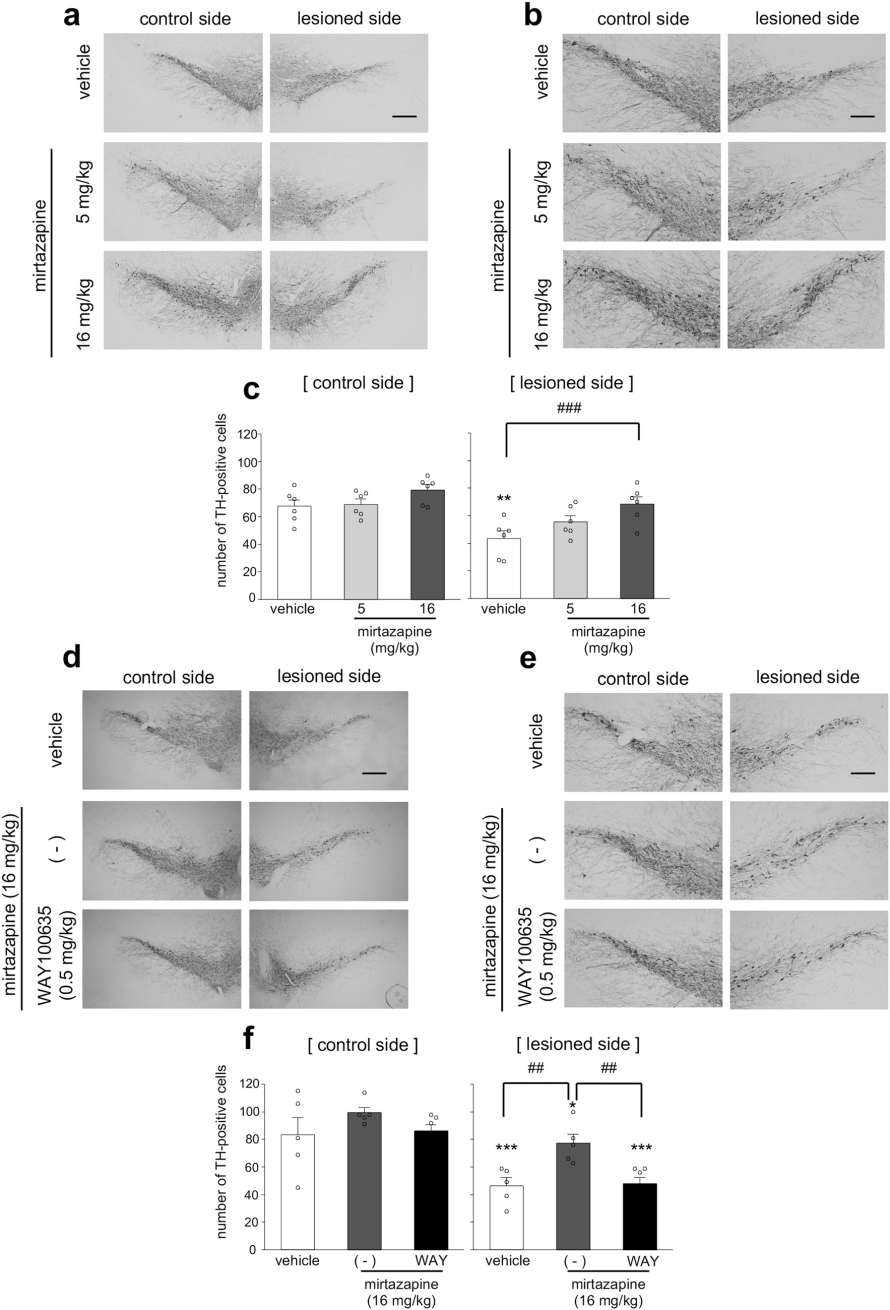
Mirtazapine administration ameliorates dopaminergic neurodegeneration via 5-HT1A receptor in parkinsonian mice. (**a**) Representative photomicrographs of TH immunohistochemistry in the SNpc of parkinsonian mice after administration of mirtazapine (5 or 16 mg/kg). Scale bar = 200 µm. (**b**) High magnification images of (**a**). Scale bar = 100 μm. (**c**) Changes in the number of TH-positive neurons after mirtazapine administration. (**d**) Representative photomicrographs of TH immunostaining in the SNpc of mirtazapine (16 mg/kg) and WAY100635 (WAY; 0.5 mg/kg) co-administered parkinsonian mice. Scale bar = 200 µm. (**e**) High magnification images of (**d**). Scale bar = 100 μm. (**f**) Quantification of the number of TH-positive cells. Data are presented as means ± SEM (n = 5–6 slices/group). *p < 0.05, **p < 0.01 and ***p < 0.001 versus control side of vehicle-treated group. ^##^ p < 0.01, ^###^p < 0.001 between the two indicated groups.

To examine whether mirtazapine could exert neuroprotective effects against dopaminergic neurodegeneration, 6-OHDA-injected parkinsonian mice were administered mirtazapine (5 or 16 mg/kg, i.p.) for 8 days. In the vehicle-treated parkinsonian mice, the number of tyrosine hydroxylase (TH)-positive dopaminergic neurons was significantly decreased in the lesioned side of the SNpc (Fig. 1a–c and supplemental Fig. 1a). Mirtazapine (16 mg/kg) treatment significantly attenuated dopaminergic neuronal loss in the lesioned side (Fig. 1a–c and supplemental Fig. 1a). To examine the involvement of 5-HT1A receptors in mirtazapine-induced dopaminergic neuroprotection, mirtazapine (16 mg/kg) were administered to parkinsonian mice with or without the 5-HT1A receptor antagonist N-[2-[4-(2-methoxyphenyl)-1-piperazinyl] ethyl]-N-2-pyridinylcyclohexanecarboxamide maleate salt (WAY100635) (0.5 mg/kg). WAY100635 treatment completely abolished the neuroprotective effects of mirtazapine (Fig. 1 d-f and supplemental Fig. 1b).

### Mirtazapine promoted astrocyte proliferation and MT-1/2 upregulation in astrocytes in the striatum of parkinsonian mice

**Figure. 2:**
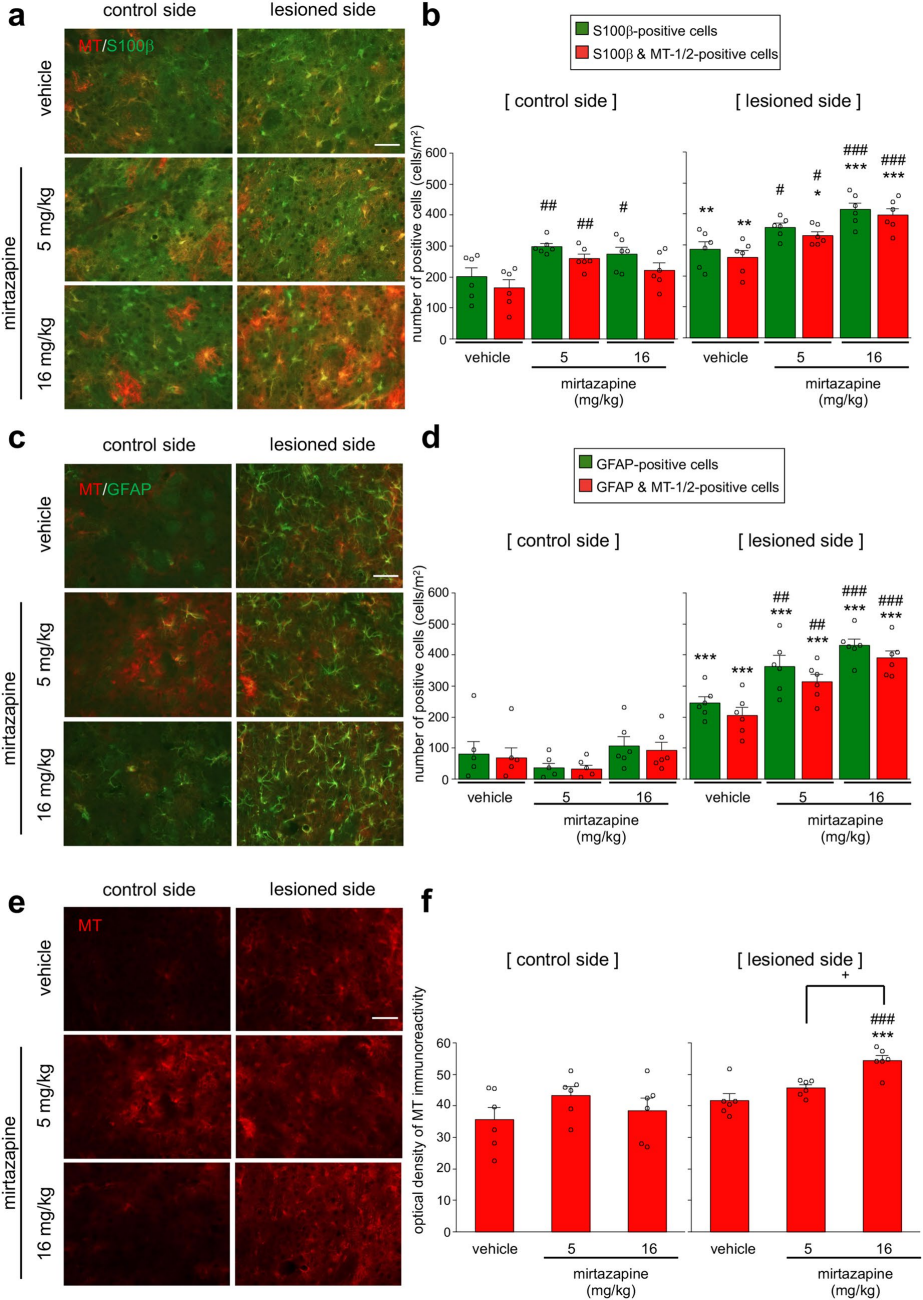
Mirtazapine administration promotes astrocyte proliferation and MT-1/2 upregulation in the striatal astrocytes of parkinsonian mice. (**a**,**c**,**e**) Representative photomicrographs of immunohistochemistry for S100β (green) and MT-1/2 (red) (**a**), GFAP (green) and MT-1/2 (red) (**c**), and MT-1/2 (red) (**e**) in the striatum of mirtazapine (5 or 16 mg/kg)-treated parkinsonian mice. Scale bar = 50 µm. (**b**) Quantification of the number of S100β- and MT-1/2-positive cells. (**d**) Quantification of the number of GFAP- and MT-1/2-positive cells. (**f**) Quantification of the optical density of MT-1/2-immunoreactivity. Data are presented as means ± SEM (n = 6). *p < 0.05, **p < 0.01, and ***p < 0.001 versus control side of each group. ^#^p < 0.05, ^##^p < 0.01, and ^###^p < 0.001 versus same side of vehicle-treated group. ^+^p < 0.05 between the two indicated groups.

Repeated administration of mirtazapine (16 mg/kg) for 8 days significantly increased MT-1/2 expression in S100β-positive astrocytes in the striatum of healthy ICR mice (Supplemental Fig. 2a,b). In the parkinsonian mice, mirtazapine treatment significantly increased the number of S100β-positive cells and MT-1/2- and S100β-positive cells in the lesioned side of the striatum (Fig. 2a,b). Furthermore, mirtazapine administration (5 and 16 mg/kg) significantly increased the number of glia fibrillary acidic protein (GFAP)-positive astrocytes and MT-1/2- and GFAP-positive cells in the lesioned side of the striatum (Fig. 2c,d). In addition, MT-1/2-immunoreactivity in the lesioned side of the striatum was also upregulated by mirtazapine (16 mg/kg) (Fig. 2e,f). In the SNpc of parkinsonian mice, mirtazapine (16 mg/kg) significantly increased the number of GFAP-positive astrocytes (Supplemental Fig. 3b) but did not significantly affect the number of MT-1/2-positive astrocytes after mirtazapine treatment (Supplemental Fig. 3a,b).

**Figure. 3:**
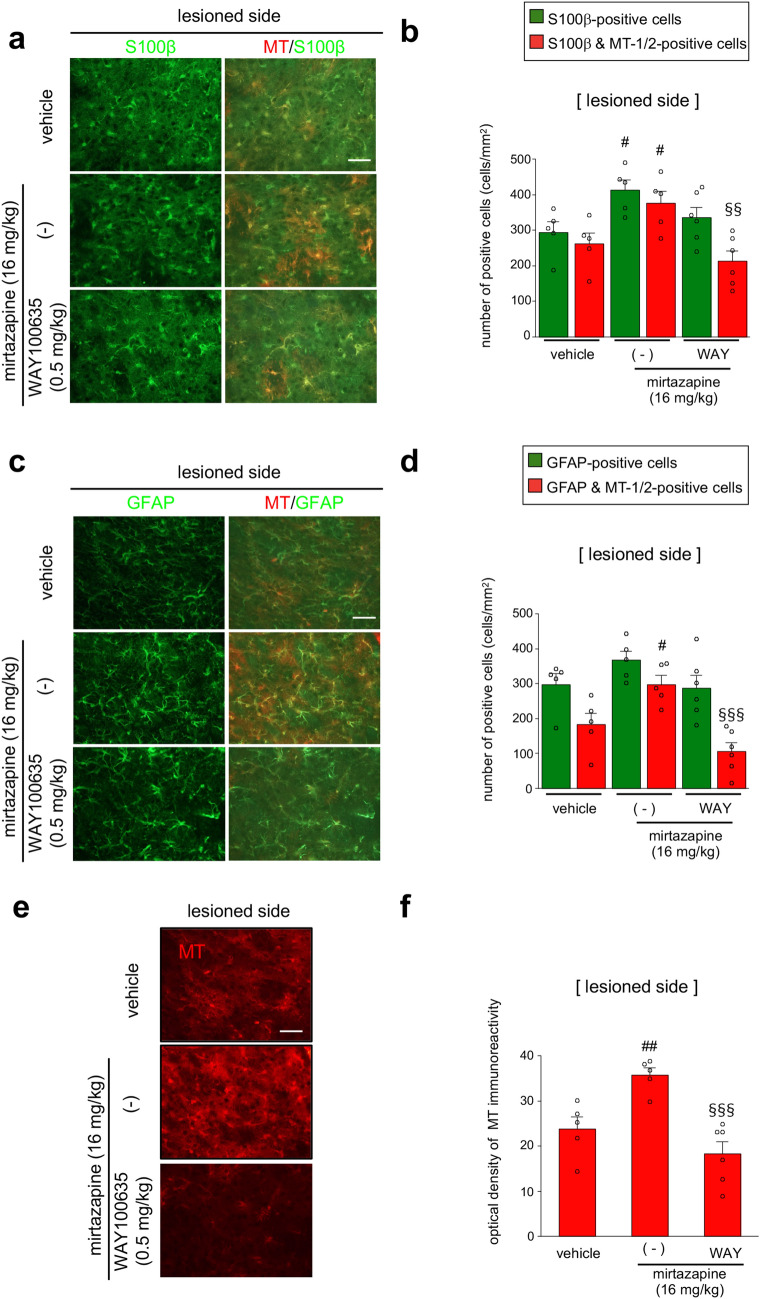
Mirtazapine promotes MT upregulation via 5-HT1A receptors. (**a**,**c**,**e**) Representative photomicrographs of immunohistochemistry for S100β (green) and MT-1/2 (red) (**a**), GFAP (green) and MT-1/2 (red) (**c**), and MT-1/2 (**e**) in the striatum of mirtazapine (16 mg/kg) and WAY100635 (WAY; 0.5 mg/kg) co-administered parkinsonian mice. Scale bar = 50 µm. (**b**) Quantification of the number of S100β- and MT-1/2-positive cells. (**d**) Quantification of the number of GFAP- and MT-1/2 positive cells. (**f**) Quantification of the optical density of MT-1/2-immunoreactivity. Data are presented as means ± SEM (n = 5–6). ^#^p < 0.05 and ^##^p < 0.01 versus lesioned side of vehicle-treated group. ^§§^p < 0.01 and ^§§§^p < 0.001 vs. mirtazapine (16 mg/kg)-treated group.

### Administration of a 5-HT1A receptor antagonist abolished mirtazapine-induced MT-1/2 upregulation

To clarify the involvement of 5-HT1A receptors in mirtazapine-induced MT-1/2 upregulation, mirtazapine (16 mg/kg) and/or WAY100635 (0.5 mg/kg) were administered to parkinsonian mice. WAY100635 treatment completely abolished mirtazapine-induced MT upregulation in S100β- (Fig. 3a,b) and GFAP-positive astrocytes (Fig. 3c,d). Increase in MT signal intensity in the lesioned side of the striatum by mirtazapine was also inhibited by WAY100635 administration (Fig. 3e,f).

### Mirtazapine protected dopaminergic neurons via 5-HT1A receptors in neuron and astrocyte cocultures but not neuronal cultures

**Figure. 4:**
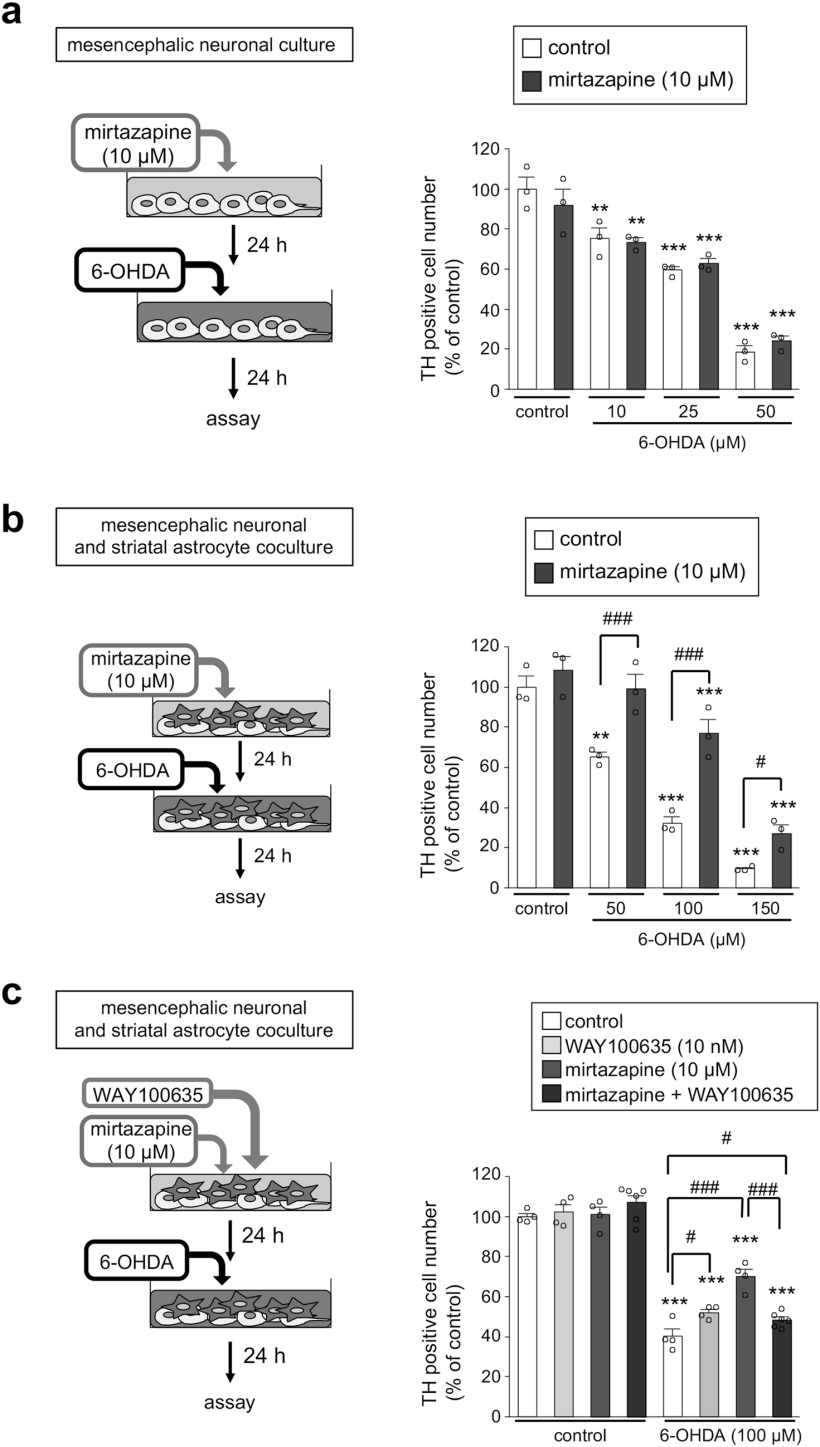
Mirtazapine requires astrocytes to exert 5-HT1A receptor-mediated dopaminergic neuroprotection. (**a**) Changes in the number of TH-positive cells in enriched neuronal cultures after treatment with mirtazapine (10 µM) followed by exposure to 6-OHDA (10–50 µM). (**b**) Changes in the number of TH-positive cells in mesencephalic neuron and striatal astrocyte cocultures after treatment with mirtazapine (10 µM) followed by exposure to 6-OHDA (50–100 µM). (**c**) Changes in the number of TH-positive cells in neuron and astrocyte cocultures after treatment with mirtazapine (10 µM) and the 5-HT1A receptor antagonist WAY100635 (10 nM) followed by exposure to 6-OHDA (100 µM). Data are presented as means ± SEM (n = 3–6) and expressed as a percentage of the control group. **p < 0.01, and ***p < 0.001 vs. each control group. ^#^p < 0.05 and ^###^p < 0.001 between the two indicated groups.

To examine the involvement of striatal astrocytes in the neuroprotective effect of mirtazapine on dopaminergic neurons, we prepared enriched mesencephalic neuronal cultures and mesencephalic neuron and striatal astrocyte cocultures. In the mesencephalic neuronal cultures, the number of TH-positive dopaminergic neurons was dose-dependently decreased by 6-OHDA exposure and pretreatment with mirtazapine did not show any neuroprotective effects against 6-OHDA-induced neurotoxicity (Fig. 4a). In contrast, in the neuron and astrocyte cocultures, mirtazapine significantly ameliorated the reduction of TH-positive neurons induced by 6-OHDA exposure (Fig. 4b). The involvement of 5-HT1A receptors in the neuroprotective effects of mirtazapine was confirmed using neuron and astrocyte cocultures. Treatment with WAY100635 completely abolished the neuroprotective effects of mirtazapine against 6-OHDA toxicity (Fig. 4c), which coincided with the results observed in parkinsonian mice (Fig. 1d–f). These results suggest that astrocytes are required for the neuroprotective effect of mirtazapine and indicate that this astrocyte-dependent neuroprotective effect is mediated via 5-HT1A receptors.

### Mirtazapine-treated neuronal conditioned medium promoted the proliferation of striatal astrocytes via astrocytic 5-HT1A receptors

**Figure. 5:**
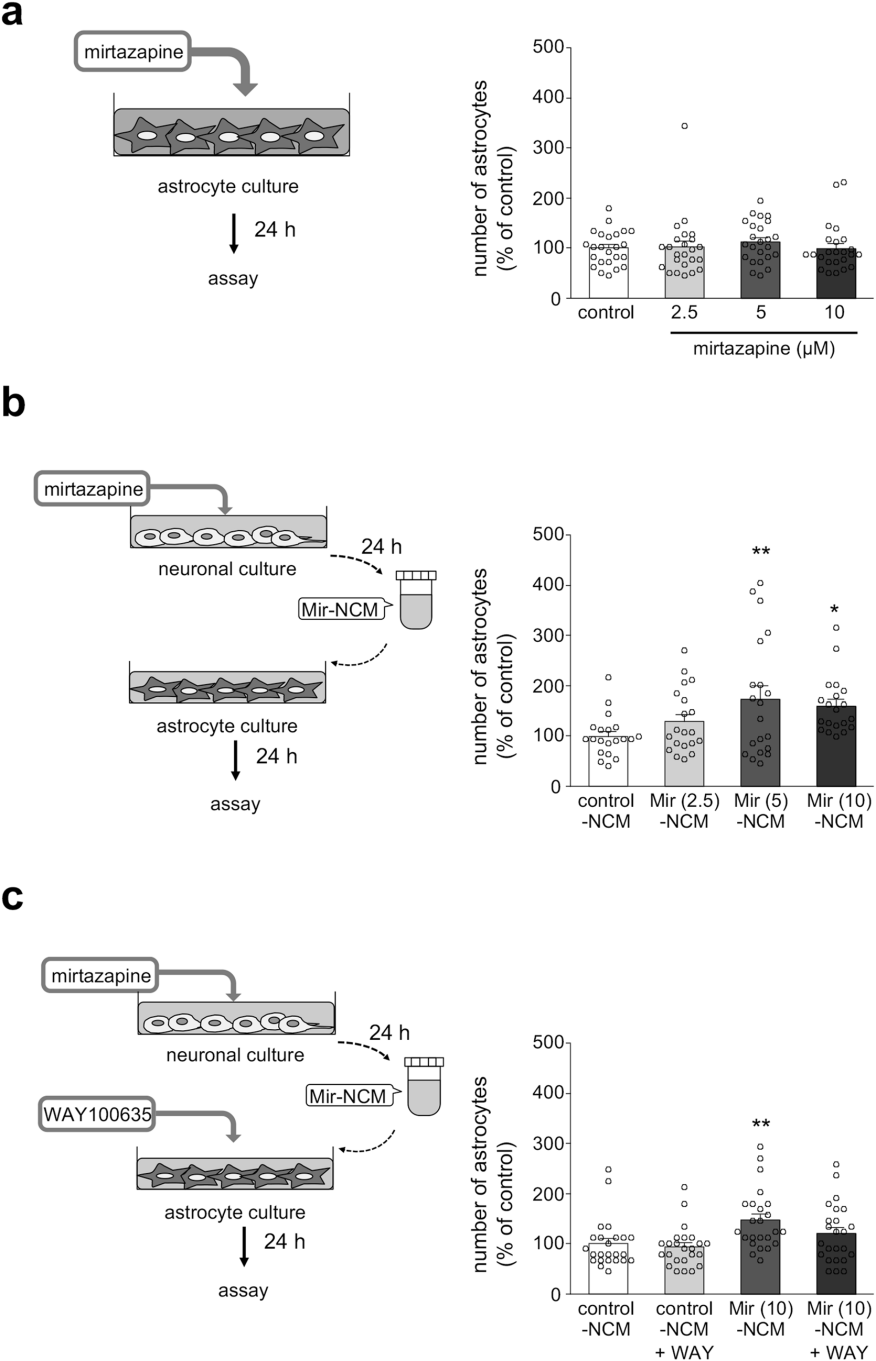
Mir-NCM promotes astrocyte proliferation via astrocytic 5-HT1A receptors. (**a**) Changes in the number of astrocytes after direct treatment with mirtazapine (2.5–10 µM) for 24 h. (**b**) Changes in the number of astrocytes after Mir (2.5–10 µM)-NCM treatment for 24 h. (**c**) Effect of WAY100635 (WAY) on the Mir-NCM-induced increase in the number of astrocytes. Astrocytes were treated with Mir-NCM (10 µM) and WAY (10 nM) for 24 h. Data are presented as means ± SEM (n = 20–24) and expressed as a percentage of the control group. *p < 0.05 and **p < 0.01 versus control-NCM treated group.

In parkinsonian mice, mirtazapine significantly increased the number of astrocytes in the lesioned side of the striatum (Fig. 2a–d). To examine whether mirtazapine enhanced astrocyte proliferation directly, primary cultured striatal astrocytes were treated with mirtazapine or neuronal conditioned medium (NCM) from mirtazapine-treated neurons (Mir-NCM). Direct treatment with mirtazapine (2.5–10 µM) for 24 h did not affect the number of astrocytes (Fig. 5a). On the other hand, Mir (5 or 10 µM)-NCM treatment significantly increased the number of astrocytes (Fig. 5b), and this increase was abolished by WAY100635 (10 nM) treatment (Fig. 5c). These results suggest that molecules secreted from mirtazapine-treated mesencephalic neurons promote astrocyte proliferation via astrocytic 5-HT1A receptors. We also measured 5-HT levels in control- and Mir-NCM using high performance liquid chromatography (HPLC). But unfortunately, we failed to detect 5-HT in cultured media, because of detection limit of HPLC (data not shown).

### Mir-NCM treatment upregulated MT-1/2 expression in striatal astrocytes via 5-HT1A receptors

**Figure. 6:**
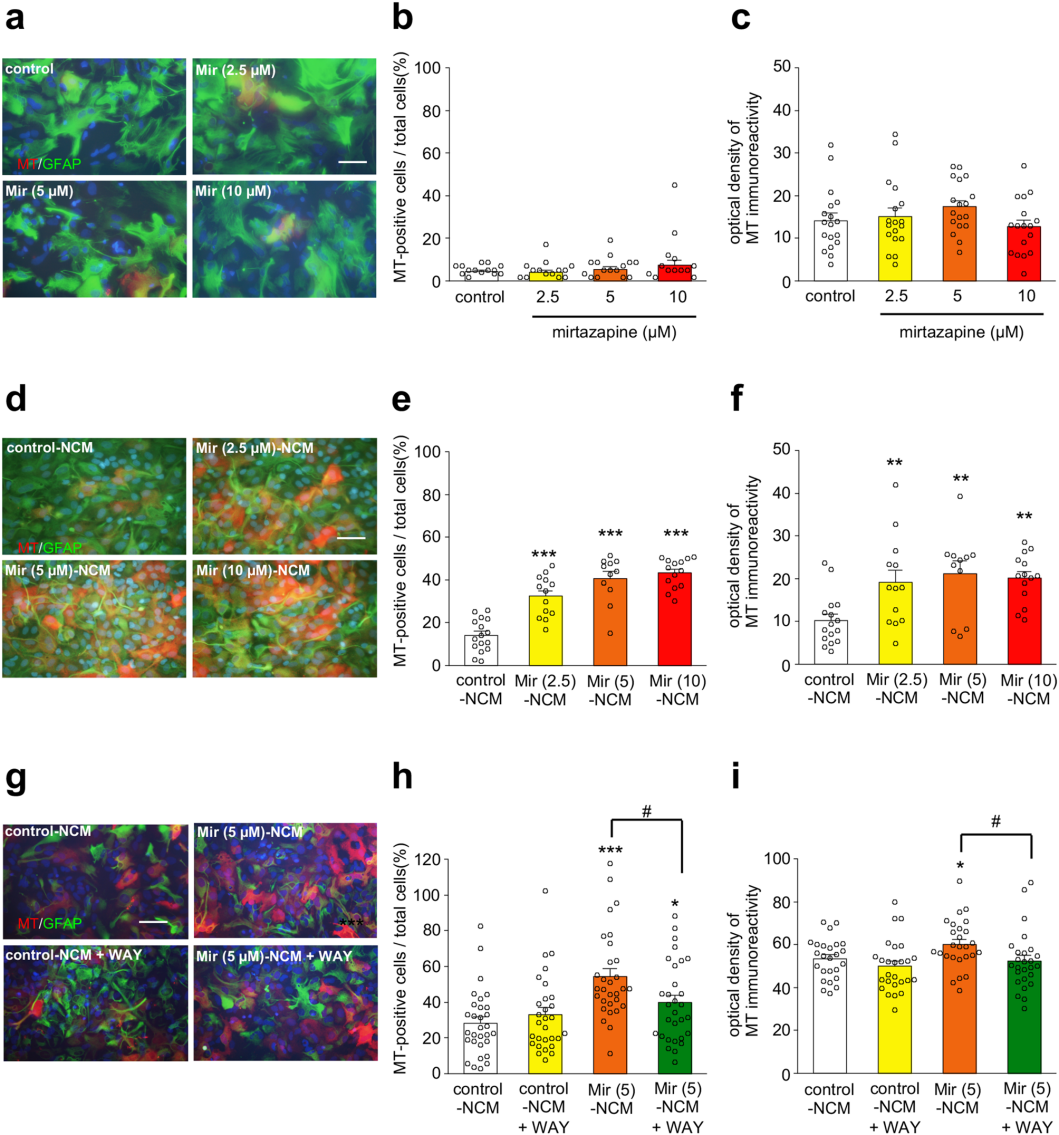
Mir-NCM upregulated MT-1/2 expression in astrocytes via 5-HT1A receptors. (**a**,**d**,**g**) Representative microphotographs of GFAP (green), MT-1/2 (red), and Hoechst (blue) staining in astrocytes treated with mirtazapine (2.5–10 µM) (**a**), Mir (2.5–10 µM)-NCM (**d**), or Mir (5 μM)-NCM + WAY100635 (WAY; 10 nM) (**g**). Scale bar = 50 µM. (**b**,**e**,**h**) The ratio of MT-positive cells to all cells (**c**,**f**,**i**) Quantification of the optical density of MT immunoreactivity. Data are presented as means ± SEM (**b**,**c**: n = 18, **e**,**f**: n = 11–16, **h**,**i**: n = 25–30). *p < 0.05, **p < 0.01 and ***p < 0.001 versus control-NCM treated group. ^#^p < 0.05 between the two indicated groups.

To examine whether mirtazapine upregulated MT-1/2 expression in astrocyte directly or through mesencephalic neurons, primary cultured striatal astrocytes were treated with mirtazapine (2.5–10 µM) or Mir (2.5–10 µM)-NCM for 24 h. Direct treatment with mirtazapine did not increase the number of MT-1/2-positive astrocytes or the signal intensity of MT-1/2 (Fig. 6a–c). On the other hand, Mir-NCM treatment significantly increased MT-1/2 expression in astrocytes (Fig. 6d–f). As shown in the in vivo experiments, administration of a 5-HT1A receptor antagonist completely abolished MT-1/2 upregulation in the striatum of the parkinsonian mice (Fig. 3a–f). Therefore, we aimed to confirm the involvement of 5-HT1A receptors in mirtazapine-induced MT-1/2 upregulation in cultured astrocytes. WAY100635 (10 nM) treatment inhibited Mir (5 µM)-NCM-induced MT-1/2 upregulation in astrocytes (Fig. 6g–i). It has been reported that MT-1/2 expression is regulated by the transcription factor nuclear factor erythroid 2-related factor 2 (Nrf2)^17^. Therefore, we examined the levels of Nrf2 and its binding activity to the antioxidant response element (ARE) in the promoter region of the rat *MT-1* gene using the nuclear fraction of astrocytes which were treated with Mir (5 µM)-NCM and/or WAY100635 (10 nM) for 3 h. In these experiments, Mir-NCM treatment failed to increase the nuclear expression or ARE-binding activity of Nrf2 (data not shown).

### Mirtazapine exerted a neuroprotective effect by promoting MT-1/2 secretion from astrocytes via 5-HT1A receptors

**Figure. 7:**
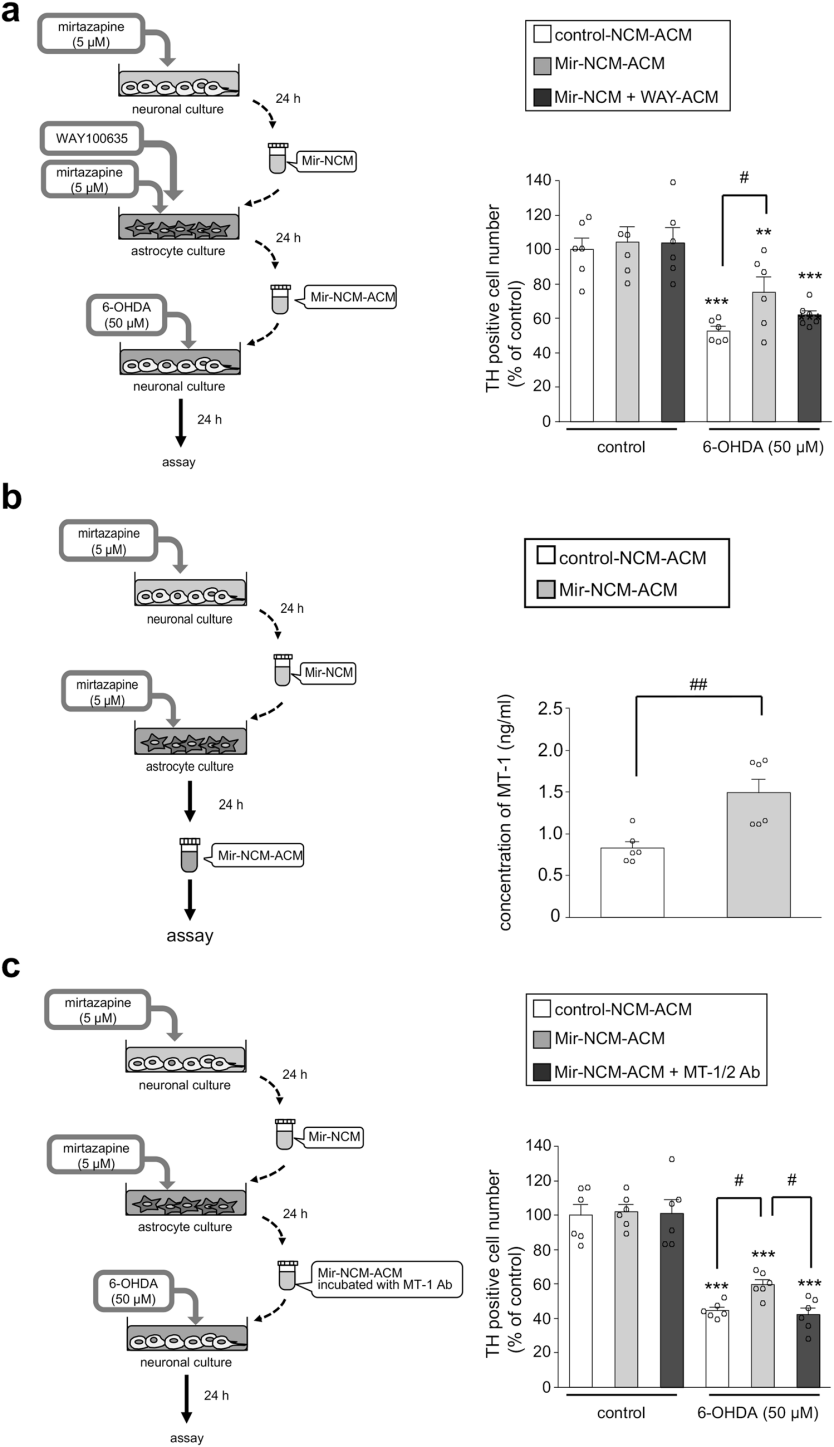
Mirtazapine protects dopaminergic neurons by modulating MT-1/2 secretion from Mir-NCM-treated astrocytes via 5-HT1A receptors. (**a**) Neuroprotective effects of mirtazapine on astrocytes. Astrocytes were treated with Mir-NCM containing fresh mirtazapine (5 µM) with or without WAY100635 (WAY; 10 nM) for 24 h. Mesencephalic neurons were treated with Mir-NCM-ACM for 24 h and then exposed to 6-OHDA (50 µM). (**b**) Concentration of MT-1 in the conditioned medium from control-NCM or Mir-NCM-treated astrocytes. Astrocytes were treated with another flesh mirtazapine-added Mir-NCM for 24 h. Data are means ± SEM (n = 6). (**c**) MT-1/2 secreted from Mir-NCM-treated astrocytes protects dopaminergic neurons against 6-OHDA-induced toxicity. To neutralize MT-1/2 in Mir-NCM-ACM, Mir-NCM-ACM was preincubated with an anti-MT-1/2 antibody for 1 h (Mir-NCM-ACM + MT-1/2 Ab), and then applied to neuronal cultures. After treatment with Mir-NCM-ACM or Mir-NCM-ACM + MT-1/2 Ab for 24 h, neuronal cultures were treated with 6-OHDA (50 µM). Data are presented as means ± SEM (n = 6) and expressed as a percentage of the control group. **p < 0.01 and ***p < 0.001 versus each control group. ^#^p < 0.05, ^##^p < 0.01 between the two indicated groups.

To examine whether secreted molecules from Mir-NCM-treated astrocytes could protected dopaminergic neurons against 6-OHDA-induced toxicity, we prepared astrocyte conditioned medium (ACM). Treatment with ACM from astrocytes, which were treated with Mir (10 µM)-NCM without mirtazapine supplementation, did not significantly prevent dopaminergic neuronal loss induced by 6-OHDA-exposure (Supplemental Fig. 4). We supposed this lack of significant dopaminergic neuroprotective effect is caused by a possible metabolism of the mirtazapine during incubation. Therefore, we treated astrocytes with another mirtazapine (5 µM) supplementation to Mir (5 µM)-NCM to fully block 5-HT2 and 3 receptors on astrocytes (Mir-NCM-ACM). This Mir-NCM-ACM significantly inhibited 6-OHDA-induced dopaminergic neuronal loss, and this neuroprotective effect was abolished by treatment with WAY100635 (Fig. 7a). Furthermore, MT-1 concentration was significantly increased in Mir-NCM-ACM (Fig. 7b). To confirm the involvement of MT-1/2 secreted from Mir-NCM-treated astrocytes in the neuroprotective effects of Mir-NCM-ACM, an anti-MT-1/2 antibody was added to Mir-NCM-ACM. The presence of the anti-MT-1/2 antibody abrogated the neuroprotective effects of Mir-NCM-ACM against 6-OHDA-induced toxicity (Fig. 7c). We also observed that mirtazapine (10 µM)-treated astrocyte conditioned medium (Mir-ACM) did not protect dopaminergic neurons from 6-OHDA-induced neurotoxicity (data not shown). These results suggest that Mir-NCM-treated astrocytes upregulate MT-1/2 expression through 5-HT1A receptors and secrete it extracellularly, which protects dopaminergic neurons against oxidative stress.

## Discussion

The present study demonstrated that mirtazapine upregulated MT-1/2 expression in striatal astrocytes and protected dopaminergic neurons in in vivo and in vitro models of PD. Furthermore, we demonstrated that mirtazapine exerts dopaminergic neuroprotection via astrocytic 5-HT1A receptors.

A previous report indicated that administration of a 16 mg/kg dose of mirtazapine improved the locomotor activity of 1-methyl-4-phenyl-1,2,3,6-tetrahydropyridine-treated parkinsonian mice^18^. Therefore, we examined the neuroprotective effects of 5 and 16 mg/kg doses of mirtazapine in this study. Our in vivo experiments showed that repeated administration of mirtazapine significantly prevented dopaminergic neurodegeneration in the SNpc of parkinsonian mice in a dose-dependent manner. However, mirtazapine treatment did not affect dopaminergic neurodegeneration in the striatum (data not shown). In our models, 6-OHDA was injected into the striatum, so we failed to observe any neuroprotective effects of mirtazapine in the striatum. Cysteine-rich MT-1/2 is known to be a strong antioxidant^19^ and is expressed predominantly in astrocytes in the central nervous system^20–22^. In our study, mirtazapine administration promoted astrocyte accumulation and MT-1/2 upregulation in the lesioned side of the striatum of parkinsonian mice. This mirtazapine-induced upregulation of MT in the striatum was also observed in healthy mice. On the other hand, mirtazapine treatment did not induce astrocytic MT expression in the SNpc. In the progression of PD, the striatal dopaminergic terminal loss precedes the neuronal loss in the SNpc^23^. Furthermore, overexpression of neurotrophic factors in striatum attenuates the loss of dopaminergic neurons in the SNpc^24^. Therefore, we suggested that mirtazapine attenuates oxidative stress in the striatum via enhancement of the MT expression in striatal astrocytes and consequently protects dopaminergic neurons in the SNpc from oxidative stress. Furthermore, administration of a 5-HT1A receptor antagonist completely abolished not only the neuroprotective effects of mirtazapine on dopaminergic neurons but also the upregulation of striatal MT-1/2 expression induced by mirtazapine. These results coincide with our previous reports^14,15^ and suggest that 5-HT1A receptor agonists could be neuroprotective drugs for PD.

Next, we explored the neuroprotective mechanism of mirtazapine using primary cultured cells. Mirtazapine exerted neuroprotective effects in neuron and astrocyte cocultures but not in neuronal cultures, and these were completely abolished by treatment with a 5-HT1A receptor antagonist. These results suggest that mirtazapine promotes astrocytes to protect dopaminergic neurons via 5-HT1A receptors. We then examined the effects of mirtazapine on the proliferation and antioxidant activity of astrocytes. Direct treatment with mirtazapine failed to induce astrocyte proliferation or MT-1/2 upregulation. On the other hand, Mir-NCM treatment significantly increased the number of astrocytes and induced MT-1/2 upregulation in astrocytes. In spite of these effects of Mir-NCM on astrocytes, treatment with Mir (10 μM)-NCN-ACM failed to protect dopaminergic neuron against 6-OHDA-induced toxicity. Mirtazapine indirectly stimulates 5-HT1A receptor though blocking 5-HT2 and 5-HT3 receptors. We supposed mirtazapine in Mir-NCM might be metabolized during incubation and fail to block 5-HT2 and 5-HT3 receptors in astrocytes, which lead to insufficient MT-1/2 expression for dopaminergic neuroprotection. To promote enough 5-HT1A receptor stimulation on astrocytes, we treated astrocytes with another mirtazapine (5 μM) supplemented Mir-NCM and obtained that media (Mir-NCM-ACM). This Mir-NCM-ACM exerted significant dopaminergic neuroprotection. These results suggested that, the both action of some released molecules from mirtazapine-treated neurons on astrocytic 5-HT1A receptors and direct effects of mirtazapine on astrocytes are involved in the neuroprotective effects of mirtazapine. Although we failed to detect 5-HT in Mir-NCM, previous report showed that mirtazapine promoted 5-HT release from serotonergic neurons and indirectly stimulates 5-HT1A receptors by blocking 5-HT2 and 5-HT3 receptors^16^. Furthermore, in the present study, the treatment with a 5-HT1A receptor antagonist reduced the Mir-NCM-induced upregulation of MT-1/2 in astrocytes and abolished the neuroprotective effects of mirtazapine. Therefore, mirtazapine may promote to release 5-HT from mesencephalic neurons by blocking α2 adrenergic receptor, and block the 5-HT2 and 5-HT3 receptors on astrocytes, which consequently stimulates 5-HT1A receptors on astrocytes. Next, we checked that Mir-NCM-ACM contained significantly higher level of MT. Furthermore, we confirmed the involvement of MT-1/2 in mirtazapine-induced neuroprotection by adding an anti-MT-1/2 antibody to Mir-NCM-ACM demonstrating that the MT-absorbed Mir-NCM-ACM failed to exert neuroprotective effects against 6-OHDA neurotoxicity. In addition to our results, it has been reported that mirtazapine increases the production of glial cell line-derived neurotrophic factor through lysophosphatidic acid 1 (LPA1) receptor in astrocytes^25^. However, in our experiments, ACM from mirtazapine-treated astrocytes could not protect dopaminergic neuron against 6-OHDA toxicity. Furthermore, LPA1 antagonist did not affect MT levels in ACM from Mir-NCM-treated astrocytes (data not shown). Take together with these facts, we suggest that mirtazapine exerts neuroprotective effects by promoting MT upregulation and secretion in/from astrocytes but not via LPA1 receptor stimulation.

Previous studies have shown that 5-HT1A receptor agonists induce the nuclear translocation of Nrf2 in astrocytes^14,15^. However, we noted no change in expression levels and ARE-binding activity of Nrf2 in the nuclei of Mir-NCM-treated astrocytes (data not shown). It has been reported that MT expression is also regulated by several factor such as nuclear factor-1 (NF-1), upstream stimulatory factor (USF-1), and metal-responsive element binding transcription factor-1 (MTF-1)^26^. These transcriptional factors might be involved in mirtazapine-induced MT expression in astrocytes. Further studies will be needed to identify the precise mechanisms of MT induction by mirtazapine.

**Figure. 8:**
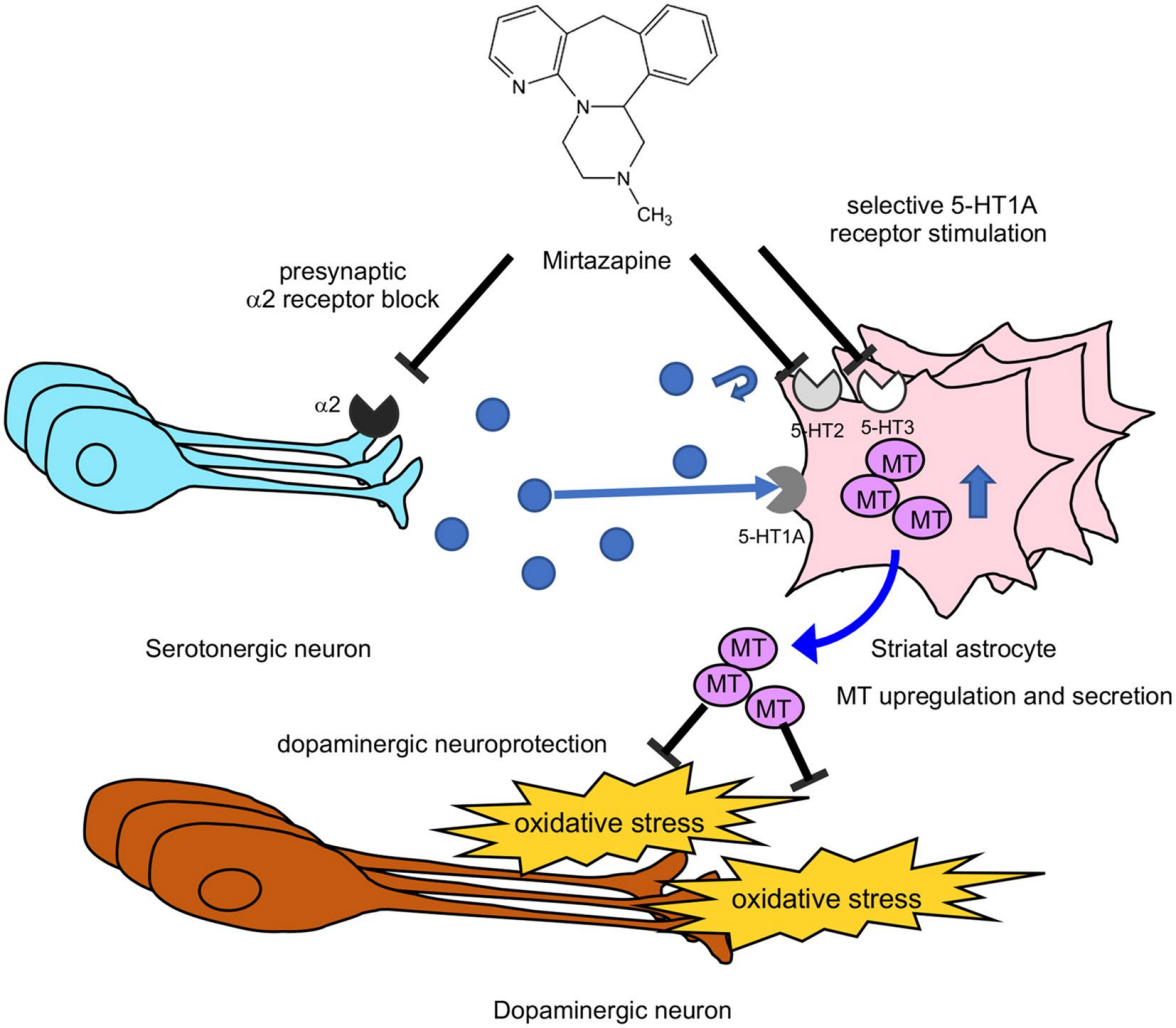
Schematic illustration of dopaminergic neuroprotective mechanisms of mirtazapine. Mirtazapine promotes the 5-HT release from serotonergic neuron by blocking adrenergic α2 receptors. Mirtazapine inhibits 5-HT2 and 5-HT3 receptor on astrocytes, which leads to selective 5-HT1A receptor stimulation and MT induction in astrocytes. MTs secreted from astrocytes attenuate oxidative stress and consequently protect dopaminergic neurons.

5-HT1A agonist improved the motor dysfunction and L-dopa-induced dyskinesia in PD model animals^27^. Furthermore, mirtazapine treatment improved motor dysfunction and non-motor symptoms such as depression and hallucination in PD patients and animal models of PD^18,28–30^. Moreover, it has been reported that mirtazapine reduces L-dopa-induced dyskinesia^31^. Evidence collected thus far suggests that mirtazapine could be a novel nosotropic and disease-modifying drug for PD. In our previous study, stimulation of astrocytic 5-HT1A receptors by 8-OH-DPAT promoted astrocyte proliferation and MT upregulation, thus protecting dopaminergic neurons^14^. In addition, we recently demonstrated that the antiparkinsonian agent rotigotine upregulated the antioxidant activity of astrocytes and exerted neuroprotection via 5-HT1A receptors in parkinsonian mice^15^. It is hypothesized that some molecules, probably 5-HT, released from mirtazapine-treated mesencephalic neurons stimulate 5-HT1A receptor on striatal astrocytes by mirtazapine-exerted 5-HT2 and -3 receptors inhibition and consequently promote MT upregulation. Released MT from astrocytes exerts neuroprotective effect on dopaminergic neurons (Fig. 8). Based on these observations, the present study demonstrates that targeting 5-HT1A receptors on astrocytes may be a possible new strategy for neuroprotection in PD.

## Methods

### Experimental animals

Male ICR mice (7 weeks old) and pregnant Sprague–Dawley (SD) rats were purchased from Charles River Japan Inc. (Yokohama, Japan). The animals were housed in a controlled environment (23 ± 1 °C, 12-h light/dark cycle) and had free access to food and water. All animal experiments were conducted in accordance with the National Institutes of Health’s Guide for the Care and Use of Experimental Animals and the Policy on the Care and Use of the Laboratory Animals of Okayama University. This study was approved by the Animal Care and Use Committee of Okayama University (approval reference number: OKU-2019103).

### Treatment of healthy ICR mice with mirtazapine

Mirtazapine (FUJIFILM Wako Pure Chemical Corporation, Osaka, Japan) was dissolved in 0.5% methylcellulose. Healthy male ICR mice weighing 38–42 g (8 weeks old) were intraperitoneally (i.p.) injected with mirtazapine (5 or 16 mg/kg) once per day for 8 days. One day after the final injection, the mice were perfused transcardially with 4% paraformaldehyde (PFA) under deep anesthesia with sodium pentobarbital (80 mg/kg, i.p.) for immunohistochemical analysis.

### Establishment of parkinsonian mice and mirtazapine treatment

Healthy male ICR mice weighing 38–42 g (8 weeks old) were anesthetized via isoflurane inhalation and placed in a stereotaxic frame (Narishige, Tokyo, Japan). All mice received unilateral injections of 6-OHDA (20 µg/site, Sigma-Aldrich, St. Louis, MO, USA) dissolved in 1 µl physiological saline containing 0.1% ascorbic acid into three areas of the right striatum. The injections were performed at the following coordinates: A + 1.2 mm, L + 2.0 mm, V + 3.0 mm; A + 0.9 mm, L + 1.4 mm, V + 3.0 mm; and A + 0.5 mm, L + 2.0 mm, V + 3.0 mm from bregma, according to the mouse brain atlas^32^. To confirm unilateral dopaminergic neurodegeneration, the mice were subcutaneously injected with apomorphine (0.5 mg/kg, Sigma Aldrich) 2 weeks after the 6-OHDA injections. Mice that exhibited rotation behavior toward the contralateral side (> 30 turns/min) were considered parkinsonian mice. One week after the apomorphine injection, the parkinsonian mice were administered mirtazapine (5 or 16 mg/kg, i.p.) once a day for 8 days. To examine the involvement of 5-HT1A receptors in the neuroprotective effects of mirtazapine, the 5-HT1A receptor antagonist WAY100635 (Sigma Aldrich) was administered (0.5 mg/kg, i.p.) 1 h before each mirtazapine injection. One day after the final injection, the mice were deeply anesthetized with pentobarbital (80 mg/kg, i.p.) and transcardially perfused with saline followed by 4% PFA for immunohistochemical analysis.

### Cell culture

Primary cultured neurons and astrocytes were prepared from the mesencephalon and striata, respectively, of SD rat embryos at 15 days of gestation using a method described previously^33^. Dissected mesencephalons and striata were cut into small pieces with scissors and incubated in 0.125% trypsin–EDTA (Thermo Fisher Scientific, Waltham, MA, USA) at 37 °C for 15 min. After centrifugation (1,500 g for 3 min), the cell pellet was treated with 0.004% DNase I (Sigma-Aldrich) containing 0.003% trypsin inhibitor (Thermo Fisher Scientific) at 37 °C for 8 min. After centrifugation (1,500 g for 3 min), the cell pellet was gently resuspended in Dulbecco’s modified Eagle medium (Invitrogen, San Diego, CA, USA) containing 10% fetal bovine serum, 4 mM L-glutamine, and 60 mg/l kanamycin sulfate (growth medium). To prepare neuronal conditioned medium, cells from the mesencephalon were plated in 6-well plates at a density of 2 × 10^5^ cells/cm^2^. To prepare mesencephalic neuronal cultures for cell viability analysis, cells from the mesencephalon were plated in 4-chamber culture slides coated with poly-D-lysine (Falcon, Corning, NY, USA) at a density of 2 × 10^4^ cells/cm^2^. Within 24 h of plating, the medium was replaced with fresh medium supplemented with 2 µM cytosine-β-D-arabinofuranoside to inhibit glial cell reproduction, and the cultures were incubated for 6 days. To obtain striatal astrocyte cultures, cells from the striata were plated in poly-D-lysine-coated 6-well plates (Falcon) at a density of 2 × 10^5^ cells/cm^2^. After incubation for 5–7 days, the cells were then subcultured. Some were seeded at a density of 3.6 × 10^4^ cells/cm^2^ in 6-well culture plates (Falcon) for extraction of the nuclear fraction or preparation of astrocyte-conditioned medium. Others were plated at a density of 2 × 10^4^ cells/cm^2^ in poly-D-lysine-coated 4-chamber culture slides (Falcon) for immunohistochemical analysis. To prepare neuron-astrocyte cocultures, astrocytes were seeded at a density of 4 × 10^4^ cells/cm^2^ directly onto mesencephalic neuronal cell layers which had been cultured in 4-chamber culture slides for 4 days. The cultures were then incubated for a further 2 days at 37 °C in a 5%-95% CO_2_-air gas mixture.

### Preparation of conditioned medium

To obtain neuronal conditioned medium (NCM), mesencephalic neurons cultured in 6-well plates were treated with mirtazapine (5 or 10 µM; Mir-NCM) or vehicle (control-NCM) for 24 h. To obtain NCM-treated astrocyte conditioned medium (ACM), striatal astrocytes cultured in 6-well plates were treated with control-NCM (control-NCM-ACM), Mir (5 μM)-NCM + mirtazapine (5 μM) (Mir-NCM-ACM) or Mir (10 μM)-NCM (Mir (10 μM)-NCM-ACM) for 24 h. To confirm the involvement of 5-HT1A receptor on astrocyte in dopaminergic neuroprotection, striatal astrocytes were treated with WAY100635 (10 nM) contained Mir-NCM (Mir-NCM + WAY-ACM). Conditioned medium were collected and centrifuged (3,000 g for 3 min) to remove cellar debris, and the supernatants were stored at -80 °C until use.

### Cell treatments

Mesencephalic neuronal cultures and mesencephalic neuron and striatal astrocyte cocultures were treated with 10 µM mirtazapine dissolved in growth medium for 24 h. After the culture medium were replaced with fresh medium, the cells were exposed to 6-OHDA (10–50 µM for neuronal cultures, 50–150 µM for neuron-astrocyte co-cultures) for 24 h. To examine the direct effects of mirtazapine on astrocyte proliferation, 1 day after subculture, astrocytes were treated with mirtazapine (2.5 5, or 10 µM) for 24 h. Moreover, to examine the direct effects of mirtazapine on MT-1/2 expression, 7 days after subculture, astrocytes were treated with mirtazapine (2.5, 5, or 10 µM) for 24 h. To examine the effects of Mir-NCM treatment on astrocyte proliferation or MT-1/2 expression, striatal astrocytes were treated with control- or Mir-NCM, respectively, for 24 h. To explore the involvement of 5-HT1A receptors in mirtazapine-induced astrocyte proliferation and MT-1/2 upregulation, astrocytes were treated with Mir-NCM with or without WAY100635 (10 nM) for 24 h. To examine whether the neuroprotective effects of mirtazapine were mediated by astrocytes, mesencephalic neurons in 4-chamber culture slides were incubated with control- or Mir-NCM-ACM for 24 h and were then exposed to 6-OHDA (50 µM) for 24 h. To examine the involvement of MT-1/2 in the neuroprotective effects of mirtazapine, Mir-NCM-ACM was preincubated with a mouse anti-MT-1/2 monoclonal antibody (DakoCytomation, Glostrup, Denmark) (1:500) for 1 h at 25 °C and then added to the neuronal cultures. After 24 h of incubation, the culture medium was replaced with fresh medium and the neurons were exposed to 6-OHDA (50 µM) for another 24 h.

### Immunohistochemistry

Immunohistochemistry was performed according to our previous reports with minor modifications^14,15^. The perfused brains were post-fixed in 4% PFA for 24 h. After cytoprotection in 15% sucrose for 48 h, the brains were frozen with powdered dry ice and cut into 20 µm-thick slices using a cryostat. For TH immunostaining of the striatum and SNpc, brain sections containing the striatum (+ 0.6 to + 1.0 mm from bregma) and SNpc (− 2.8 to − 3.0 mm from bregma) were treated with 0.5% H_2_O_2_ for 30 min at 25 °C. After they were blocked with 1% normal goat serum in 10 mM PBS containing 0.2% Triton X-100 (0.2% PBST) for 30 min, the slices were incubated with a rabbit anti-TH polyclonal antibody (1:2,000; Merck Millipore, Burlington, MA, USA) for 18 h at 4 °C. After the slices were washed, they were then incubated with a goat biotinylated anti-rabbit IgG secondary antibody (1:1,000; Vector Laboratories, Burlingame, CA, USA) for 2 h at 25 °C followed by an avidin–biotin peroxidase complex (1:2,000; Vector Laboratories) for 1 h at 25 °C. TH-immunopositive signals were visualized via a reaction with 3,3′-diaminobenzidine, nickel, and H_2_O_2._ To examine MT expression in astrocytes, striatal sections were soaked in 1% normal goat serum in 0.2% PBST for 30 min and incubated with a mouse anti-MT-1/2 monoclonal antibody (1:500; DakoCytomation), GFAP polyclonal antibody (1:5,000; DakoCytomation), or rabbit anti-S100β polyclonal antibody (1:5,000; DakoCytomation) for 18 h at 4 °C. After they were washed, the slices were incubated with an Alexa Fluor 594-conjugated goat anti-mouse IgG or Alexa Fluor 488-conjugated goat anti-rabbit IgG secondary antibody (1:1,000; Invitrogen) in 0.2% PBST for 2 h at 25 °C.

Cells on 4-chamber culture slides were fixed with 4% PFA for 30 min at 25 °C. They were then blocked with 2.5% normal goat serum in 10 mM PBS containing 0.1% Triton-X-100 (0.1% PBST) for 20 min and incubated with a mouse anti-TH monoclonal antibody (1:1,000; Millipore), mouse anti-MT-1/2 monoclonal antibody (1:50; DakoCytomation), rabbit anti-GFAP polyclonal antibody (1:2,000, DakoCytomation), or rabbit anti-GFAP polyclonal antibody (1:2,000; Novus Biologicals, Littleton, CO, USA) for 18 h at 4 °C. After they were washed three times with 10 mM PBS (10 min each), the cells were incubated with an Alexa Fluor 594-conjugated goat anti-mouse IgG secondary antibody or Alexa Fluor 488-conjugated goat anti-rabbit IgG secondary antibody (1:1,000; Invitrogen) for 1.5 h. The cells were then counterstained with Hoechst 33342 nuclear stain.

All slides were analyzed under a fluorescence microscope (Olympus BX53, Tokyo, Japan) and the images collected were analyzed with cellSens imaging software (v1.16, Olympus). A mercury lamp with a 470–495 nm, 530–550 nm, or 360–370 nm band-pass filter to excite Alexa Fluor 488, Alexa Fluor 594, or Hoechst dye, respectively. Light emitted from Alexa Fluor 488, Alexa Fluor 594, or Hoechst was collected through a 510–550 nm band-pass filter, 590 nm long-pass filter, or 420 nm long-pass filter, respectively. Adobe Photoshop CS4 software (v11.0) was used for digital amplification of the image.

### Enzyme-linked immunosorbent assay (ELISA)

Levels of MT-1 in control-NCM-ACM and Mir-NCM-ACM were measured using mouse MT-1 ELISA kit (SEB199Ra; Cloud-Clone Corp., Katy, TX, USA) according to the manufacture’s protocol.

### High-performance liquid chromatography (HPLC)

To measure 5-HT concentration in control- and Mir-NCM HPLC analysis was performed according to previous reports^34^. Briefly, HPLC system was consisted of an EP-300 liquid chromatography pump (EP-700 MK; Eicom, Kyoto, Japan), a DGU-4A degasser (DG-300; Eicom), a reversed-phase octadecyl silica (ODS) column (Eicompak SC-5ODS, 3.0 ϕ × 150 mm; Eicom), and an ECD-300 electrochemical detector (+ 750 mV relative to the Ag/AgCl reference electrode; Eicom). The mobile phase was composed of an acetic-citrate buffer (pH 3.5) which contained 200 mg/l sodium 1-octanesulfonate, 5 mg/l ethylenediaminetetraacetic acid and 15% methanol. Mobile phase was degassed and pumped at a flow rate of 0.5 ml/min. Peaks were recorded using a PowerChrom integrator (Eicom).

### Quantitative analysis

Quantitative analyses were also performed according to our previous studies^14,15^. The number of TH-positive neurons in the SNpc was counted under a microscope at 100× magnification. The boundary between the SNpc and ventral tegmental area was defined by a line extending dorsally from the most medial boundary of the cerebral peduncle. The number of GFAP-, S100β-, or MT-1/2-immunopositive cells in the striatum was counted manually under a microscope at 200× magnification and the ratio of MT-positive cells to GFAP- or S100β-positive cells was calculated. The intensity of MT-1/2-immunopositive signals in the striatum was measured using ImageJ version 1.44k (National Institutes of Health, Bethesda, MD, USA). The number of TH-positive neurons in the neuronal cultures was counted under a microscope in all areas of each chamber slide. For astrocyte proliferation analysis, cultured astrocytes were counterstained with Hoechst 33342 nuclear stain and the number of cells was counted in six to ten randomly chosen fields/wells under a microscope at 400× magnification. The numbers of MT-1/2- and GFAP-immunopositive cells in the astrocyte cultures were counted in six to ten randomly chosen fields under a microscope at 400× magnification. These results were expressed as the ratio of MT-1/2-immunopositive cells to all cells. The immunoreactivity of MT-1/2 was measured using ImageJ.

### Statistical analysis

All data are expressed as means ± SEM. Statistical analyses were performed using Kaleida Graph software (v4.0, Hulinks Inc., Tokyo, Japan). Comparisons between multiple groups were performed using one-way ANOVA followed by Fisher’s least significant difference test. A *p*-value < 0.05 was considered statistically significant. All of statistical data are shown in supplemental table.

## Supplementary information

Supplementary figures.

Supplementary Dataset.

## Data Availability

All data are available from the authors upon reasonable request.
