# Supplementary material for: Mirtazapine exerts astrocyte-mediated dopaminergic neuroprotection
Source: Sci Rep. 2020 Nov 26;10:20698. doi: 10.1038/s41598-020-77652-4 (PMC7693322; doi:10.1038/s41598-020-77652-4)
Supplement: Supplementary file 1 — Supplementary figures. [file 41598_2020_77652_MOESM1_ESM.pdf]

Revised Supplementary Files

Revised supplementary file1: revised supplementary figures 1-4
Revised supplementary file2: revised supplementary dataset

Mirtazapine exerts astrocyte-mediated dopaminergic neuroprotection

Ryo Kikuoka[1], Ikuko Miyazaki[1], Natsuki Kubota[1], Megumi Maeda[1], Daiki Kagawa[1], Masaaki Moriyama[1], Asuka Sato[1], Shinki Murakami[1], Yoshihisa Kitamura[2], Toshiaki Sendo[2], Masato Asanuma[1, *]

[1] *Department of Medical Neurobiology, Okayama University Graduate School of Medicine, Dentistry and Pharmaceutical Sciences, Okayama, Japan*
[1] *Department of Clinical Pharmacy, Okayama University Graduate School of Medicine, Dentistry and Pharmaceutical Sciences, Okayama, Japan*

*Corresponding author:
Prof. Masato Asanuma, MD, PhD,
Department of Medical Neurobiology, Okayama University Graduate School of Medicine, Dentistry and Pharmaceutical Sciences
2-5-1 Shikata-cho, Kita-ku, Okayama, 700-8558, Japan
Tel: +81-86-235-7096

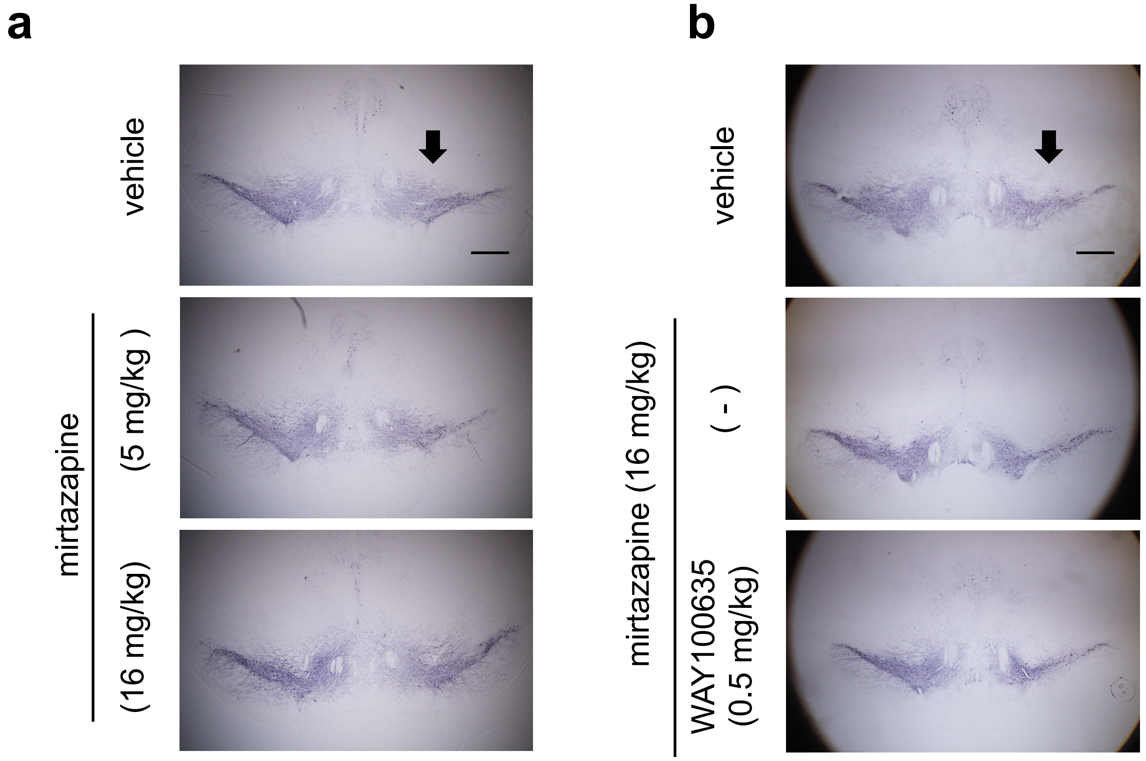

Supplemental Figure 1. Representative microphotographs of immunohistochemistry for TH in the SNpc of hemi-PD. (a) Mirtazapine (5-16 mg/kg)-treated mice. (b) Mirtazapine (16 mg/kg) with or without WAY 100635 (0.5 mg/kg)-treated mice. Right side is the 6-OHDA-injcted lesioned side (arrow). Scale bar = 500 µm.

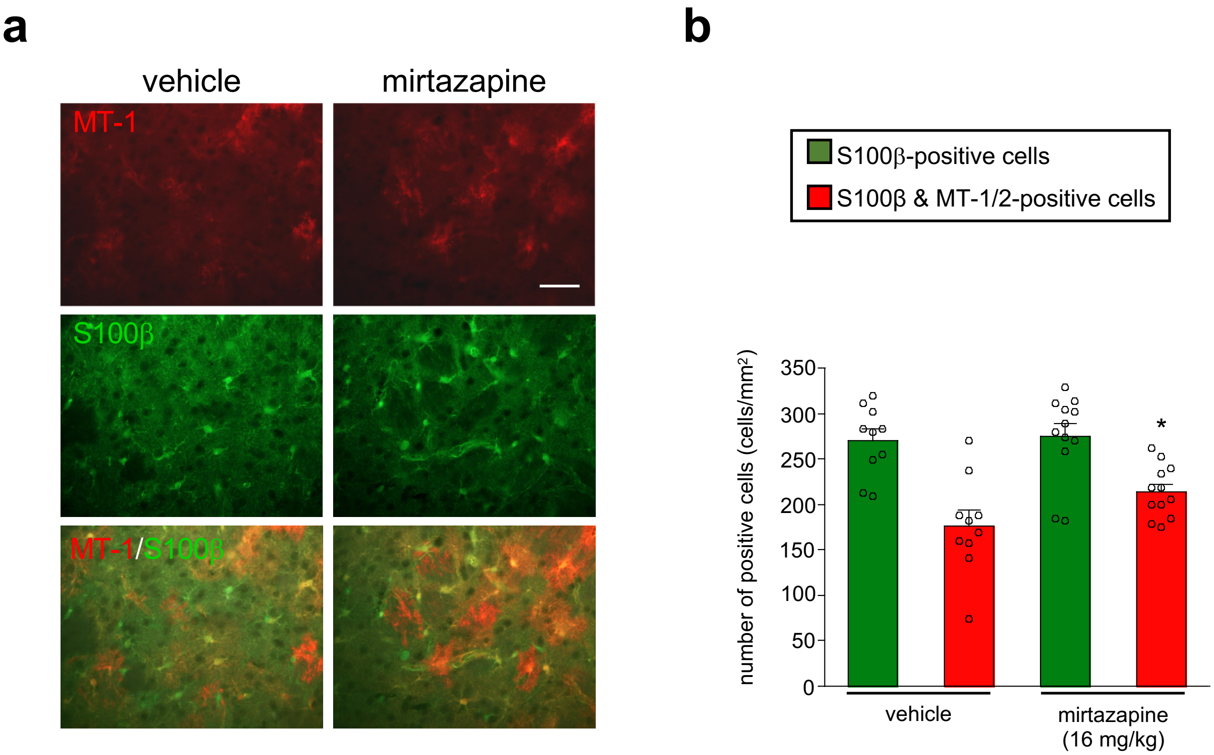

Supplemental Figure 2. Mirtazapine upregulated MT-1/2 expression in the striatal astrocytes of healthy ICR mice. (a) Representative microphotograph of immunohistochemistry for S100β (green) and MT-1/2 (red) in the striatum of mirtazapine (16 mg/kg)-treated mice. Scale bar = 50 μm. (b) Quantitation of the S100β- and MT-1/2-positive cells. Data are means ± SEM (n = 5-6). *$p < 0.05$ vs. vehicle-treated control group.

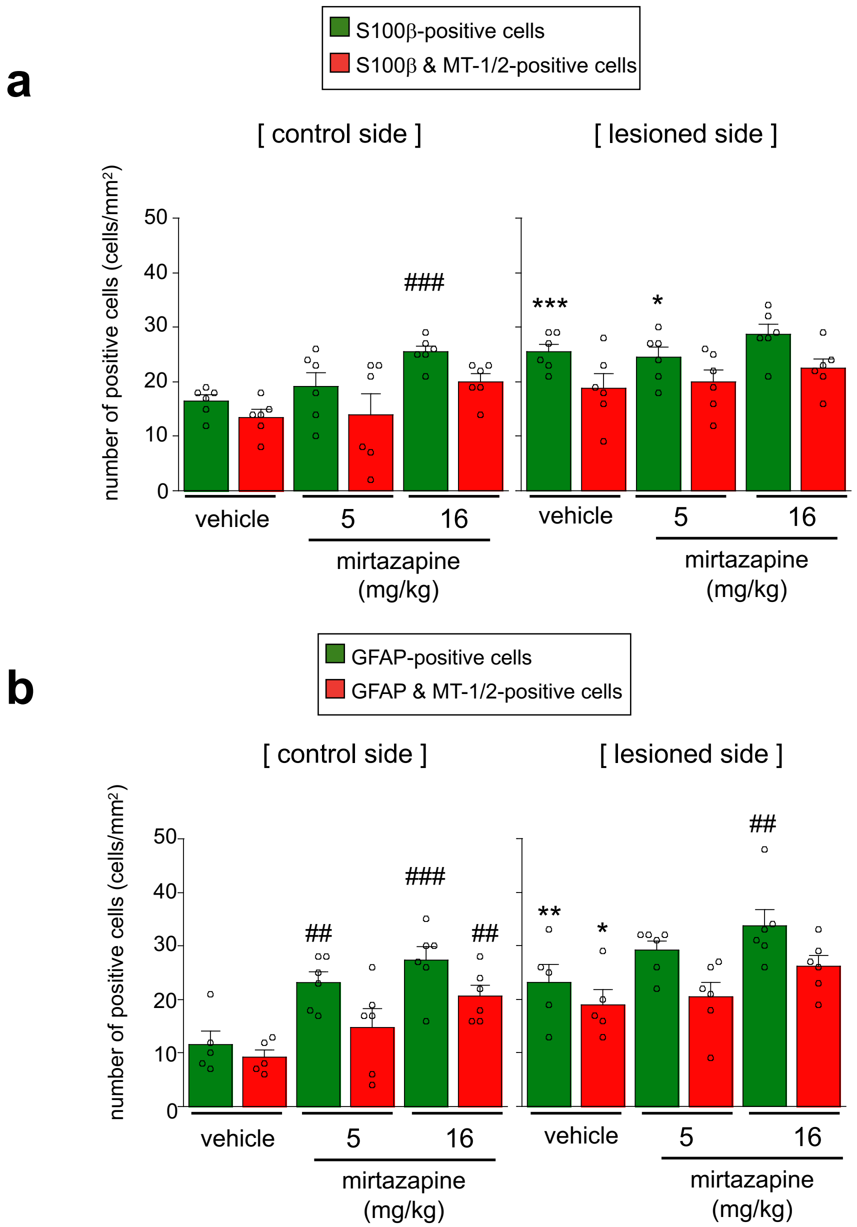

Supplemental Figure 3. Effects of mirtazapine administration on MT-1/2 expression in astrocytes in the SNpc of parkinsonian mice. (a) Quantification of the S100β - and MT-1/2-positive cells. (b) Quantitation of the GFAP- and MT-1/2-positive cells. Data are means ± SEM (n = 5-6). *$p < 0.05$, **$p < 0.01$ vs. control side of each group. $^{##}p < 0.01$, $^{###}p < 0.001$ vs. the same side of vehicle-treated group.

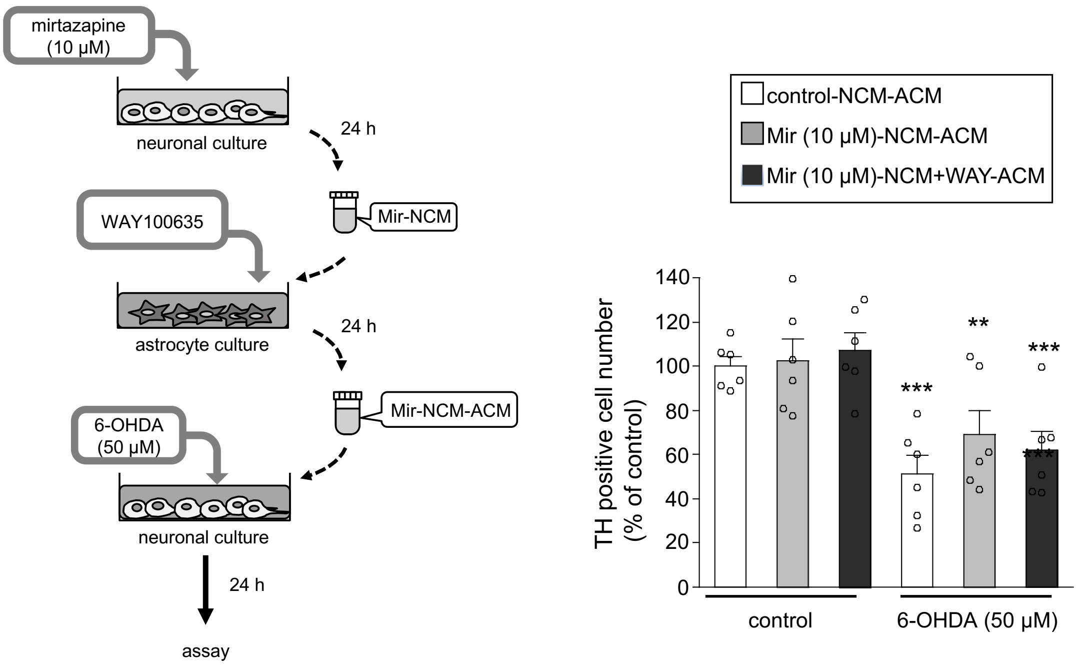

Supplemental Figure 4. Changes in the number of TH-positive cells in neuronal cultures after treatment with Mir (10 µM)-NCM-ACM with or without WAY100635, followed by 6-OHDA exposure. To prepare Mir-NCM-ACM, astrocytes were treated with mirtazapine (10 µM)-treated-NCM for 24 h. Mesencephalic neurons were pre-treated with Mir-NCM-ACM for 24 h, and then exposed to 6-OHDA (50 µM) for 24 h. Data are means ± SEM (n = 6) expressed as percentage of control group. **$p < 0.01$, ***$p < 0.001$ vs. each control groups.
