# Supplementary material for: Mirtazapine exerts astrocyte-mediated dopaminergic neuroprotection
Source: Sci Rep. 2020 Nov 26;10:20698. doi: 10.1038/s41598-020-77652-4 (PMC7693322; doi:10.1038/s41598-020-77652-4)
Supplement: Supplementary file 2 — Supplementary Dataset. [file 41598_2020_77652_MOESM2_ESM.pdf]

Revised Supplementary Files

Revised supplementary file1: revised supplementary figures 1-4
Revised supplementary file2: revised supplementary dataset

Mirtazapine exerts astrocyte-mediated dopaminergic neuroprotection

Ryo Kikuoka[1], Ikuko Miyazaki[1], Natsuki Kubota[1], Megumi Maeda[1], Daiki Kagawa[1], Masaaki Moriyama[1], Asuka Sato[1], Shinki Murakami[1], Yoshihisa Kitamura[2], Toshiaki Sendo[2], Masato Asanuma[1, *]

[1] *Department of Medical Neurobiology, Okayama University Graduate School of Medicine, Dentistry and Pharmaceutical Sciences, Okayama, Japan*
[1] *Department of Clinical Pharmacy, Okayama University Graduate School of Medicine, Dentistry and Pharmaceutical Sciences, Okayama, Japan*

*Corresponding author:
Prof. Masato Asanuma, MD, PhD,
Department of Medical Neurobiology, Okayama University Graduate School of Medicine, Dentistry and Pharmaceutical Sciences
2-5-1 Shikata-cho, Kita-ku, Okayama, 700-8558, Japan
Tel: +81-86-235-7096

Figure number		group	n	P value	F/t Values	Test type
1c	TH positive cells			p = 0.00015	F (5,35) = 7.241	one-way ANOVA followed by post-hoc Fisher's LSD test
	control side	vehicle	6			
		mirtazapine (5 mg/kg)	6	p = 0.821 (vs. same side of vehicle-treated group)		
		mirtazapine (16 mg/kg)	6	p = 0.0819 (vs. same side of vehicle-treated group)		
	lesioned side	vehicle	6	p = 0.001 (vs. control side of each group)		
		mirtazapine (5 mg/kg)	6	p = 0.0741 (vs. same side of vehicle-treated group)<br>p = 0.0515 (vs. control side of each group)		
		mirtazapine (16 mg/kg)	6	p = 0.0007 (vs. same side of vehicle-treated group)<br>p = 0.0603 (vs. same side of mirtazapine (5 mg/kg)-treated group)<br>p = 0.1908 (vs. control side of each group)		
1f	TH			p < 0.0001	F (5,31) = 10.339	one-way ANOVA followed by post-hoc Fisher's LSD test
	control side	vehicle	5			
		mirtazapine	5	p = 0.1108(vs. same side of vehicle-treated group)		
		mirtazapine + WAY	6	p = 0.165 (vs. same side of vehicle-treated group)		
	lesioned side	vehicle	5	p = 0.0009 (vs. control side of each group)		
		mirtazapine	5	p = 0.0042 (vs. same side of vehicle-treated group)<br>p = 0.0323 (vs. control side of each group)		
		mirtazapine + WAY	6	p = 0.879 (vs. same side of vehicle-treated group)<br>p = 0.0043 (vs. same side of mirtazapine-treated group)<br>p = 0.0002 (vs. control side of each group)		
2b	S100β			p < 0.0001	F (5,35) = 12.377	one-way ANOVA followed by post-hoc Fisher's LSD test
	control side	vehicle	6			
		mirtazapine (5 mg/kg)	6	p = 0.0026 (vs. same side of vehicle-treated group)		
		mirtazapine (16 mg/kg)	6	p = 0.0181 (vs. same side of vehicle-treated group)		
	lesioned side	vehicle	6	p = 0.0072 (vs .control side of each group)		
		mirtazapine (5 mg/kg)	6	p = 0.023 (vs. same side of vehicle-treated group)<br>p = 0.0554 (vs. control side of each group)		
		mirtazapine (16 mg/kg)	6	p = 0.0001 (vs. same side of vehicle-treated group)<br>p < 0.0001 (vs. control side of each group)		
	S100β & MT			p < 0.0001	F (5,35) = 16.075	
	control side	vehicle	6			
		mirtazapine (5 mg/kg)	6	p = 0.0025 (vs. same side of vehicle-treated group)		
		mirtazapine (16 mg/kg)	6	p = 0.0581 (vs. same side of vehicle-treated group)		
	lesioned side	vehicle	6	p = 0.0024 (vs. control side of each group)		
		mirtazapine (5 mg/kg)	6	p = 0.021 (vs. same side of vehicle-treated group)<br>p = 0.0202 (vs. control side of each group)		
		mirtazapine (16 mg/kg)	6	p < 0.0001 (vs. same side of vehicle-treated group)<br>p < 0.0001 (vs. control side of each group)		
2d	GFAP			p < 0.0001	F (5,35) = 32.815	one-way ANOVA followed by post-hoc Fisher's LSD test
	control side	vehicle	6			
		mirtazapine (5 mg/kg)	6	p = 0.273 (vs. same side of vehicle-treated group)		
		mirtazapine (16 mg/kg)	6	p = 0.501 (vs. same side of vehicle-treated group)		
	lesioned side	vehicle	6	p = 0.0003 (vs. control side of each group)		
		mirtazapine (5 mg/kg)	6	p = 0.0065 (vs. same side of vehicle-treated group)<br>p < 0.0001 (vs. control side of each group)		
		mirtazapine (16 mg/kg)	6	p < 0.0001 (vs. same side of vehicle-treated group)<br>p < 0.0001 (vs. control side of each group)		
	GFAP & MT			p < 0.0001	F (5,35) = 32.884	
	control side	vehicle	6			
		mirtazapine (5 mg/kg)	6	p = 0.3295 (vs. same side of vehicle-treated group)		
		mirtazapine (16 mg/kg)	6	p = 0.495 (vs. same side of vehicle-treated group)		
	lesioned side	vehicle	6	p = 0.0005 (vs. control side of each group)		
		mirtazapine (5 mg/kg)	6	p = 0.005 (vs. same side of vehicle-treated group)<br>p < 0.0001 (vs. control side of each group)		
		mirtazapine (16 mg/kg)	6	p < 0.0001 (vs. same side of vehicle-treated group)<br>p < 0.0001 (vs. control side of each group)		
2f	MT density			p = 0.00089	F (5,35) = 5.629	one-way ANOVA followed by post-hoc Fisher's LSD test
	control side	vehicle	6			
		mirtazapine (5 mg/kg)	6	p = 0.0554 (vs. same side of vehicle-treated group)		
		mirtazapine (16 mg/kg)	6	p = 0.458 (vs. same side of vehicle-treated group)		
	lesioned side	vehicle	6	p = 0.13 (vs. control side of each group)		
		mirtazapine (5 mg/kg)	6	p = 0.315 (vs. same side of vehicle-treated group)<br>p = 0.564 (vs. control side of each group)		
		mirtazapine (16 mg/kg)	6	p = 0.00027 (vs. same side of vehicle-treated group)<br>p = 0.0003 (vs. control side of each group)<br>p = 0.0318 (vs. same side of mirtazapine (5 mg/kg)-treated group)		
3b	S100β			p = 0.0406	F (2,15) = 4.14	one-way ANOVA followed by post-hoc Fisher's LSD test
		vehicle	5			
		mirtazapine	5	p = 0.0142 (vs. vehicle-treated group)		
		mirtazapine + WAY	6	p = 0.317 (vs. vehicle-treated group)<br>p = 0.0774 (vs. mirtazapine-treated group)		
	S100β & MT			p = 0.00717	F (2,15) = 7.395	one-way ANOVA followed by post-hoc Fisher's LSD test
		vehicle	5			
		mirtazapine	5	p = 0.0249 (vs. vehicle-treated group)		
		mirtazapine + WAY	6	p = 0.274 (vs. vehicle-treated group)<br>p = 0.0022 (vs. mirtazapine-treated group)		
3d	GFAP			p = 0.207	F (2,15) = 1.776	one-way ANOVA followed by post-hoc Fisher's LSD test
		vehicle	5			
		mirtazapine	5			
		mirtazapine + WAY	6			
	GFAP & MT			p = 0.00109	F (2,15) = 12.073	one-way ANOVA followed by post-hoc Fisher's LSD test
		vehicle	5			
		mirtazapine	5	p = 0.0141 (vs. vehicle-treated group)		
		mirtazapine + WAY	6	p = 0.0738 (vs. vehicle-treated group)<br>p = 0.0003 (vs. mirtazapine-treated group)		
3f	MT density			p = 0.00067	F (2,15) = 13.496	one-way ANOVA followed by post-hoc Fisher's LSD test
		vehicle	5			
		mirtazapine	5	p = 0.005 (vs. vehicle-treated group)		
		mirtazapine + WAY	6	p = 0.132 (vs. vehicle-treated group)<br>p = 0.0002 (vs. mirtazapine-treated group)		

4a	TH positive cells			p < 0.0001	F (7,23) = 43.986	one-way ANOVA followed by post-hoc Fisher's LSD test
	control	6-OHDA (0 μM)	3			
		6-OHDA (10 μM)	3	p = 0.0011 (vs. 6-OHDA (0 μM) group)		
		6-OHDA (25 μM)	3	p < 0.0001 (vs. 6-OHDA (0 μM) group)		
		6-OHDA (50 μM)	3	p < 0.0001 (vs. 6-OHDA (0 μM) group)		
	Mirtazapine (10 μM)	6-OHDA (0 μM)	3			
		6-OHDA (10 μM)	3	p = 0.009 (vs. Mirtazapine (10 μM)+6-OHDA (0 μM) group)<br>p = 0.756 (vs. control+6-OHDA (10 μM) group)		
		6-OHDA (25 μM)	3	p = 0.0002 (vs. Mirtazapien (10μM)+6-OHDA (0 μM) group)<br>p = 0.599 (vs. control+6-OHDA (25 μM) group)		
		6-OHDA (50 μM)	3	p < 0.0001 (vs. Mirtazapine (10 μM)+6-OHDA (0 μM) group)<br>p = 0.404 (vs. control+6-OHDA (50 μM) group)		
4b	TH positive cells			p < 0.0001	F (7,23) = 54.075	one-way ANOVA followed by post-hoc Fisher's LSD test
	control	6-OHDA (0 μM)	3			
		6-OHDA (50 μM)	3	p = 0.002 (vs. 6-OHDA (0 μM) group)		
		6-OHDA (100 μM)	3	p < 0.0001 (vs. 6-OHDA (0 μM) group)		
		6-OHDA (150 μM)	3	p < 0.0001 (vs. 6-OHDA (0 μM) group)		
	Mirtazapine (10 μM)	6-OHDA (0 μM)	3			
		6-OHDA (50 μM)	3	p = 0.225 (vs. Mirtazapine (10μM)+6-OHDA (0μM) group)<br>p = 0.0003 (vs. control+6-OHDA (50 μM) group)		
		6-OHDA (100 μM)	3	p = 0.0005 (vs. Mirtazapine (10 μM)+6-OHDA (0 μM) group)<br>p < 0.0001 (vs. control+6-OHDA (100 μM) group)		
		6-OHDA (150 μM)	3	p < 0.0001 (vs. Mirtazapine (10μM)+6-OHDA (0 μM) group)<br>p = 0.0275 (vs. control+6-OHDA (150 μM) group)		
4c	TH positive cells			p < 0.0001	F (7,35) = 93.897	one-way ANOVA followed by post-hoc Fisher's LSD test
	control	control	4			
		WAY100635 (10 nM)	4	p = 0.612 (vs. control+6-OHDA (0 μM) group)		
		Mirtazapine (10 μM)	4	p = 0.795 (vs. control+6-OHDA (0 μM) group)		
		WAY100635 + mirtazapine	6	p = 0.936 (vs. control+6-OHDA (0 μM) group)		
	6-OHDA (100 μM)	control	4	p < 0.0001 (vs. control+6-OHDA (0 μM) group)		
		WAY100635 (10 nM)	4	p < 0.0001 (vs. WAY+6-OHDA (0μM) group)<br>p = 0.0108 (vs. control+6-OHDA (100 μM) group)<br>p = 0.0003 (vs. Mirtazapine (10 μM)+6-OHDA (100 μM) group)		
		Mirtazapine (10 μM)	4	p < 0.0001 (vs. Mirtazapine (10 μM)+6-OHDA (0 μM) group)<br>p < 0.0001 (vs. control+6-OHDA (100 μM) group)		
		WAY100635 + mirtazapine	6	p < 0.0001 (vs. WAY + mirtazapine (10μM)-6OHDA (0μM) group)<br>p = 0.0479 (vs. control+6-OHDA (100 μM) group)<br>p < 0.0001 (vs. Mirtazapine (10 μM)+6-OHDA (100 μM) group)		
5a	number of astrocyte			p = 0.714	F (3,95) = 0.454	one-way ANOVA followed by post-hoc Fisher's LSD test
		control	24			
		Mirtazapine (2.5 μM)	24			
		Mirtazapine (5 μM)	24			
		Mirtazapine (10 μM)	24			
5b	number of astrocyte			p = 0.0144	F (7,79) = 3.747	one-way ANOVA followed by post-hoc Fisher's LSD test
		control-NCM	20			
		Mir (2.5 μM)-NCM	20	p = 0.227 (vs. control-NCM group)		
		Mir (5 μM)-NCM	20	p = 0.003 (vs. control-NCM group)		
		Mir (10 μM)-NCM	20	p = 0.0147 (vs. control-NCM group)		
5c	number of astrocyte			p = 0.00297	F (3,95) = 4.992	one-way ANOVA followed by post-hoc Fisher's LSD test
		control-NCM	24			
		control-NCM + WAY	24	p = 0.678 (vs. control-NCM group)		
		Mir (10 μM)-NCM	24	p = 0.0026 (vs. control-NCM group)		
		Mir (10 μM)-NCM + WAY	24	p = 0.188 (vs. control-NCM group)<br>p = 0.0804 (vs. Mir-NCM group)		
6b	number of MT-immunopositive cell			p = 0.47	F (3,71) = 0.851	one-way ANOVA followed by post-hoc Fisher's LSD test
		control	18			
		Mirtazapine (2.5 μM)	18			
		Mirtazapine (5 μM)	18			
		Mirtazapine (10 μM)	18			
6c	MT density			p = 0.7	F (3,71) = 0.475	one-way ANOVA followed by post-hoc Fisher's LSD test
		control	18			
		Mirtazapine (2.5 μM)	18			
		Mirtazapine (5 μM)	18			
		Mirtazapine (10 μM)	18			
6e	number of MT-immunopositive cell			p < 0.0001	F (3,53) = 33.796	one-way ANOVA followed by post-hoc Fisher's LSD test
		control-NCM	16			
		Mir (2.5 μM)-NCM	13	p < 0.0001 (vs. control-NCM group)		
		Mir (5 μM)-NCM	11	p < 0.0001 (vs. control-NCM group)		
		Mir (10 μM)-NCM	14	p < 0.0001 (vs. control-NCM group)		
6f	MT density			p = 0.00178	F (3,53) = 5.785	one-way ANOVA followed by post-hoc Fisher's LSD test
		control-NCM	16			
		Mir (2.5 μM)-NCM	13	p = 0.0046 (vs. control-NCM group)		
		Mir (5 μM)-NCM	11	p = 0.0011 (vs. control-NCM group)		
		Mir (10 μM)-NCM	14	p = 0.0014 (vs. control-NCM group)		
6h	number of MT-immunopositive cell			p < 0.0001	F (3,118) = 8.177	one-way ANOVA followed by post-hoc Fisher's LSD test
		control-NCM	30			
		control-NCM + WAY	29	p = 0.401 (vs. control-NCM group)		
		Mir (5 μM)-NCM	30	p < 0.0001 (vs. control-NCM group)		
		Mir (5 μM)-NCM + WAY	30	p = 0.0419 (vs. control-NCM group)<br>p = 0.0107 (vs. Mir-NCM group)		
6i	MT density			p = 0.0159	F (3,102) = 3.609	one-way ANOVA followed by post-hoc Fisher's LSD test
		control-NCM + control	25			
		control-NCM + WAY	26	p = 0.307 (vs.control-NCM group)		
		Mir (5 μM)-NCM + control	26	p = 0.0406 (vs. control-NCM group)		
		Mir (5 μM)-NCM + WAY	26	p = 0.763 (vs. control-NCM group)<br>p = 0.0182 (vs. Mir-NCM group)		

7a	TH positive cells			p < 0.0001	F (5,35) = 10.88	one-way ANOVA followed by post-hoc Fisher's LSD test
	control	control-NCM-ACM	6			
		Mir-NCM-ACM	6	p = 0.666 (vs. control-NCM-ACM+6-OHDA (0 μM) group)		
		Mir-NCM + WAY100635-ACM	6	p = 0.688 (vs. control-NCM-ACM+6-OHDA (0 μM) group)		
	6-OHDA (50 μM)	control-NCM-ACM	6	p < 0.0001 (vs. control-NCM-ACM+6-OHDA(0 μM) group)		
		Mir-NCM-ACM	6	p = 0.0061 (vs. Mir-NCM-ACM+6-OHDA (0μM) group)		
				p = 0.0273 (vs. control-NCM-ACM+6-OHDA (50 μM) group)		
				p = 0.0002 (vs. Mir-NCM + WAY-ACM+6-OHDA (0 μM) group)		
		Mir-NCM + WAY100635-ACM	6	p = 0.358 (vs. control-NCM-ACM+6-OHDA (50 μM) group)		
				p = 0.175 (vs. Mir-NCM-ACM+6-OHDA (50 μM) group)		
7b	MT-1 concentration			p = 0.00403	F (1,11) = 13.782	one-way ANOVA followed by post-hoc Fisher's LSD test
		control-NCM-ACM	6			
		Mir-NCM-ACM	6	p = 0.004 (vs. control-NCM-ACM)		
7c	TH positive cells			p < 0.0001	F (5,35) = 36.334	one-way ANOVA followed by post-hoc Fisher's LSD test
	control	control-NCM-ACM	6			
		Mir-NCM-ACM	6	p = 0.758 (vs. control-NCM-ACM+6-OHDA (0 μM) group)		
		Mir-NCM-ACM + MT-1/2 Ab	6	p = 0.874 (vs. control-NCM-ACM+6-OHDA (0 μM) group)		
	6-OHDA (50 μM)	control-NCM-ACM	6	p < 0.0001 (vs. control-NCM-ACM+6-OHDA (0 μM) group)		
				p < 0.0001 (vs. Mir-NCM-ACM+6-OHDA (0μM) group)		
		Mir-NCM-ACM	6	p = 0.0347 (vs. control-NCM-ACM+6-OHDA (50 μM) group)		
				p < 0.0001 (vs. Mir-NCM-ACM + MT-1/2 Ab+6-OHDA (0 μM) group)		
		Mir-NCM-ACM + MT-1/2 Ab	6	p = 0.745 (vs. control-NCM-ACM+6-OHDA (50 μM) group)		
				p = 0.0165 (vs. Mir-NCM-ACM+6-OHDA (50 μM) group)		

Figure number		group	n	P value	F/t Values	Test type
supplemental fig. 2b	S100β			p = 0.815	F (1,21) = 0.0558	one-way ANOVA followed by post-hoc Fisher's LSD test
		vehicle	5			
		mirtazapine (16 mg/kg)	6			
	S100β & MT			p = 0.0469	F (1,21) = 4.4483	one-way ANOVA followed by post-hoc Fisher's LSD test
		vehicle	5			
		mirtazapine (16 mg/kg)	6	p = 0.0469 (vs. vehicle-treated group)		
supplemental fig. 3a	S100β			p = 0.00016	F (5,35) = 7.173	one-way ANOVA followed by post-hoc Fisher's LSD test
	control side	vehicle	6			
		mirtazapine (5 mg/kg)	6	p = 0.275 (vs. same side of vehicle-treated group)		
		mirtazapine (16 mg/kg)	6	p = 0.0008 (vs. same side of vehicle-treated group)		
	lesioned side	vehicle	6	p = 0.0008 (vs. control side of each group)		
		mirtazapine (5 mg/kg)	6	p = 0.679 (vs. same side of vehicle-treated group)<br>p = 0.0339 (vs. control side of each group)		
		mirtazapine (16 mg/kg)	6	p = 0.196 (vs. same side of vehicle-treated group)<br>p = 0.196 (vs. control side of each group)		
	S100β & MT			p = 0.0651	F (5,35) = 2.347	one-way ANOVA followed by post-hoc Fisher's LSD test
	control side	vehicle	6			
		mirtazapine (5 mg/kg)	6			
		mirtazapine (16 mg/kg)	6			
	lesioned side	vehicle	6			
		mirtazapine (5 mg/kg)	6			
		mirtazapine (16 mg/kg)	6			
supplemental fig. 3b	GFAP			p < 0.0001	F (5,33) = 8.141	one-way ANOVA followed by post-hoc Fisher's LSD test
	control side	vehicle	6			
		mirtazapine (5 mg/kg)	6	p = 0.0041 (vs. same side of vehicle-treated group)		
		mirtazapine (16 mg/kg)	6	p = 0.0002 (vs. same side of vehicle-treated group)		
	lesioned side	vehicle	6	p = 0.0056 (vs. control side of each group)		
		mirtazapine (5 mg/kg)	6	p = 0.118 (vs. same side of vehicle-treated group)<br>p = 0.1 (vs. control side of each group)		
		mirtazapine (16 mg/kg)	6	p = 0.0086 (vs. same side of vehicle-treated group)<br>p = 0.0835 (vs. control side of each group)		
	GFAP & MT			p = 0.00163	F (5,33) = 5.23	one-way ANOVA followed by post-hoc Fisher's LSD test
	control side	vehicle	6			
		mirtazapine (5 mg/kg)	6	p = 0.127 (vs. same side of vehicle-treated group)		
		mirtazapine (16 mg/kg)	6	p = 0.0034 (vs. same side of vehicle-treated group)		
	lesioned side	vehicle	6	p = 0.0142 (vs. control side of each group)		
		mirtazapine (5 mg/kg)	6	p = 0.679 (vs. same side of vehicle-treated group)<br>p = 0.108 (vs. control side of each group)		
		mirtazapine (16 mg/kg)	6	p = 0.0556 (vs. same side of vehicle-treated group)<br>p = 0.119 (vs. control side of each group)		
supplemental fig. 4				p < 0.0001	F (5,35) = 7.96	one-way ANOVA followed by post-hoc Fisher's LSD test
	control	control-NCM-ACM	6			
		Mir(10 μM)-NCM-ACM	6	p = 0.836 (vs. control-NCM-ACM+6-OHDA (0 μM) group)		
		Mir(10 μM)-NCM + WAY100635-ACM	6	p = 0.559 (vs. control-NCM-ACM+6-OHDA (0 μM) group)		
	6-OHDA (50 μM)	control-NCM-ACM	6	p = 0.0003 (vs. control-NCM-ACM+6-OHDA (0 μM) group)		
		Mir(10 μM)-NCM-ACM	6	p = 0.0096 (vs. Mir (10 μM)-NCM-ACM+6-OHDA (0 μM) group)<br>p = 0.148 (vs. control-NCM-ACM+6-OHDA (50μM) group)		
		Mir(10 μM)-NCM + WAY100635-ACM	6	p = 0.0007 (vs. Mir(10 μM)-NCM + WAY-ACM+6-OHDA (0 μM) group)<br>p = 0.544 (vs. Mir (10 μM)-NCM-ACM+6-OHDA (50 μM) group)		
